# Development of a general method for detection and quantification of the P35S promoter based on assessment of existing methods

**DOI:** 10.1038/srep07358

**Published:** 2014-12-08

**Authors:** Yuhua Wu, Yulei Wang, Jun Li, Wei Li, Li Zhang, Yunjing Li, Xiaofei Li, Jun Li, Li Zhu, Gang Wu

**Affiliations:** 1 Key Laboratory of Oil Crop Biology of the Ministry of Agriculture, Oil Crops Research Institute, Chinese Academy of Agricultural Sciences, No. 2 Xudong 2nd Road, Wuhan 430062, People's Republic of China; 2 College of Life Sciences, Hubei University, No. 368 Friendship Avenue, Wuhan 430062, People's Republic of China; 3 Supervision and Test Center (Wuhan) for Environmental Safety of Genetically Modified Plants, Ministry of Agriculture, No. 2 Xudong 2nd Road, Wuhan 430062, People's Republic of China; 4 School of Life Science, South-Central University for Nationalities, Min-Yuan Road 708, Wuhan 430074, People's Republic of China

## Abstract

The *Cauliflower mosaic virus* (CaMV) 35S promoter (P35S) is a commonly used target for detection of genetically modified organisms (GMOs). There are currently 24 reported detection methods, targeting different regions of the P35S promoter. Initial assessment revealed that due to the absence of primer binding sites in the P35S sequence, 19 of the 24 reported methods failed to detect P35S in MON88913 cotton, and the other two methods could only be applied to certain GMOs. The rest three reported methods were not suitable for measurement of P35S in some testing events, because SNPs in binding sites of the primer/probe would result in abnormal amplification plots and poor linear regression parameters. In this study, we discovered a conserved region in the P35S sequence through sequencing of P35S promoters from multiple transgenic events, and developed new qualitative and quantitative detection systems targeting this conserved region. The qualitative PCR could detect the P35S promoter in 23 unique GMO events with high specificity and sensitivity. The quantitative method was suitable for measurement of P35S promoter, exhibiting good agreement between the amount of template and Ct values for each testing event. This study provides a general P35S screening method, with greater coverage than existing methods.

Ever since the first genetically modified (GM) crop was commercially planted, genetically modified organisms (GMOs) have come under suspicion from governments and citizens because of potential safety risks^1^,^2^. Many countries have stipulated legislation to regulate GMOs and GMO-derived products. The core of GMO regulation involves detecting GMOs, analyzing legality of their components in a particular region, and determining the need for labeling. Thus GMO detection technology is the requisite of GMO safety management.

Polymerase chain reaction (PCR) is the most generally accepted GMO detection technique. This is largely because of its ability to amplify specific DNA fragments from highly processed materials^2^. PCR-based GMO detection strategies include element screening, construct-specific, and transgenic event-specific methods^3^. The construct-specific detection method involves targeting the junction between two elements, and it is not able to distinguish two different events transformed with the same plasmid^4^. Event-specific detection can precisely distinguish legitimate transgenic events from related illegal varieties transformed with similar or identical transgenic constructs, thus it is often used to evaluate the legality of a GMO sample^3^. The screening method targets the most frequently used elements in transgenic constructs, has the lowest specificity, and is mainly used for rapid evaluation of high numbers of GMOs. In 2013, 336 GM crop varieties from 27 different species were commercialized worldwide^5^. This number is rising as ever more GM crops in the research stage enter field trials, in which, only partial varieties were developed event-specific detection methods. In general, it is not feasible to conduct PCR tests for all possible events during GMO detection. A common practice is to begin with general screening of a small number of targets common to numerous events, such as the *Cauliflower mosaic virus* (CaMV) 35S RNA gene promoter (P35S) and the terminator of the *Agrobacterium tumefaciens* Ti plasmid nopaline synthase gene (TNOS)^6^,^7^,^8^,^9^.

Using the available GMO transformation information from the GM Crop Database (http://cera-gmc.org/index.php?action=gm_crop_database), we conducted preliminary statistical analyses into the presence of P35S and TNOS in GM crops. This revealed that 65.7% (67/102) of approved commercial GM events contain the P35S promoter, 53.49% (55/102) the NOS terminator, and 81.4 (83/102) either or both of these in their transgene constructs (Supplementary Table S1 online). In commercially important transgenic crops, the presence percentage of these two components is higher. Among the 28 commercial events of transgenic maize (*Zea mays*), only one (LY038; Monsanto, St. Louis, MO, USA) contained neither element, and in 16 commercial GMOs of rapeseed (*Brassica napus*) there were only two exceptions. Some transgenic events, such as GM maize Bt11, T25, and GM cotton Mon531 contained two copies of P35S in their transforming constructs. Consequently, the P35S promoter and NOS terminator are the most widely used GMO screening targets.

Because of the importance of the P35S promoter in screening detection of GMOs, a large variety of GMO screening tests have been established and published^10^,^11^,^12^,^13^,^14^,^15^,^16^,^17^,^18^,^19^,^20^,^21^,^22^,^23^,^24^,^25^,^26^,^27^,^28^,^29^,^30^,^31^,^32^,^33^,^34^,^35^,^36^,^37^,^38^,^39^,^40^,^41^,^42^,^43^,^44^,^45^. Some of these methods have been adopted by ISO, EU, China, and other countries and regions as standard methods for GMO detection^10^,^11^,^13^,^14^,^15^,^16^,^28^,^31^,^32^,^33^,^34^,^45^. The GMO Detection Method Database (GMDD) developed by Shanghai Jiao Tung University collected 37 methods from literature, ISO standards, and Chinese standards. These include 21 qualitative methods, 14 quantitative methods, and two methods used for microarray analysis (http://gmdd.shgmo.org/). In the GMDD, some primer pairs are simultaneously used by both qualitative and quantitative assays, and some methods are actually repeats of the same method using different primer names. The P35S promoter is one of the most frequently modified elements in GMOs. The P35S promoter sequences in different GMOs and vectors may be different from each other owing to origination from different strains or from modification in vector construction or mutation during the breeding process^13^. Previous oligonucleotide comparison revealed differences among P35S promoter sequences from the CaMV genome, event 176, Bt11, T25, MON810, and DLL25^17^. Morisett *et al*. identified a single nucleotide polymorphism (SNP) in the primer binding site of the P35S sequence in TC1507 maize, leading to inefficient amplification of testing primer/probe sets^9^,^46^.

Although many P35S-based methods are available for the testing laboratories, only partial methods have gone through necessary validation processes and inter-laboratory studies against a small number of transgenic events^13^,^14^,^19^,^24^,^31^,^34^,^43^,^45^. Indeed, no one method has been systematically verified for accuracy and sensitivity across all commercially available transgenic events. The International Life Science Institute (ILSI) petitioned over 100 laboratories to survey the use of P35S and TNOS for the detection of GMOs. Some laboratories encountered methodological flaws of P35S in their testing, such as low sensitivity, low reproducibility, and false positives or negatives^9^. Holden *et al*. tested the suitability of five published P35S-based methods with eight maize reference materials, demonstrating that two methods had the flaws of poor linear regression parameters and multiple PCR amplicons in some of the testing materials^9^.

The human immunodeficiency virus (HIV) often generates drug resistance mutations owing to highly variable gene and drug selection pressure. During detection of HIV-1 drug resistance mutations, the genotyping assay, combining reverse transcription PCR with sequencing technology or high resolution melting (HRM) analysis, is commonly used to detect all the possible mutations in HIV genome^47^,^48^, the design of RT-PCR primers should target conserved regions flanking mutational hot spots, and the used primers must be specific to the region of interest. Similarly, the primers for GMO detection also should lie within conserved regions and be specific to the target sequence, and the detection of P35S requires amplification of a conserved region across different transgene events. Otherwise, the P35S-based methods would exhibit the above flaws, even result in false testing results during GMO screening.

P35S-based methodologies play an important role during the GMO screening phase. Currently, GMO detection laboratories select methods from different sources, including published literature, ISO standards, databases, or in-house developed methods. This heterogeneity in methodology may result in divergent test results if the P35S sequence carried by the testing sample was altered during construction of the transforming vector or in the breeding process. The ILSI survey revealed that most participating laboratories were interested in adopting a standardized method, which could generate consistent testing results and lead to better inter-laboratory reproducibility^9^. Currently, no optimal method is available that is based on the comprehensive comparison of the existing methods.

The purpose of this study was (1) to isolate the P35S sequence from different transgenic events, (2) to analyze methodological flaws in existing P35S-based detection methods using sequence alignment between primers/probes and the P35S sequences from different GMOs and constructs, and to confirm the defects found by the experiments, and (3) to design a general qualitative and quantitative detection system that targets the conserved region of P35S for high-coverage GMO screening.

## Results

### Sequence alignment

A total of 67 GM events containing P35S have been collected by the GM crop database (http://www.cera-gmc.org/GMCropDatabase), of those, 23 unique GM events were available in the present study. After performing isolation of P35S promoters, nineteen P35S sequences were isolated from the following 16 transgenic events: GM soybean GTS-40-3-2, A5547-127; GM maize MON863, NK603, TC1507, Bt11, MON810, T25; GM cotton MON15985, MON88913, MON531, LLCotton 25, MON1445; GM rapeseed OXY235; and GM rice Kefeng 6 and KMD. The isolated sequences were submitted to the GenBank database, and accession numbers and sequences are summarized in Table 1. The P35S sequence length varied among the transgenic events, ranging from 307 bp in MON 810 to 1385 bp in LLCotton 25. Three transgenic events (Bt11, T25, and MON531) had two copies of P35S in their transforming constructs, with the sequence of these two copies different from each other for each event (Table 1).

Twelve P35S promoter fragments were collected: four maize events MON88017, MON89034, 59122, and 98140 (carrying three copies of P35S) from the patent database^49^,^50^,^51^,^52^; two rapeseed events Topas 19/2 and T45 from the application dossier; one rice event LLRice62 and three commonly used transgenic binary vectors pBI121, pCambia-1381, and pMCG161 from the GenBank database (Table 1). Both isolated and downloaded P35S sequences were aligned with the whole genome of CaMV (NC_001497.1) using the bl2seq program in NCBI, to determine the relative position of the P35S promoters in the CaMV genome. The homologous region and the SNP number of each P35S compared to CaMV genome are given in Table 1. In transgenic events selected for this study, all of the isolated P35S promoters normally drive the target genes to express functional proteins. We therefore concluded that all P35S sequences were complete. In accordance with the results of the sequence alignment, we obtained the conserved region of P35S across different transgenic events, corresponding to the genomic region of CaMV between positions 7148 and 7342. The sequence alignment results revealed that 13 P35S sequences, from events A5547-127, NK603, MON810, T25, 59122, MON88017, 98140, MON15985, MON88913, MON531 and LLRice62, exhibited 100% identity with the CaMV genomic sequence, while the other P35S sequences exhibited different degrees of variation (Table 1). For the conserved regions, 13 P35S sequences from GTS-40-3-2, MON863, TC1507, Bt11 (two copies), MON89034, MON531, LLcotton 25, MON1445, Kefeng 6, KMD, pCambia-1381, and pMCG161 showed differences from the CaMV genomic sequence, with homologies from 93% to 99%, the other collected P35S sequences showed 100% identity with the CaMV genomic sequence (Table 1). The sequence comparison also revealed that seven P35S promoters, from NK603, MON88017, MON15985, MON88913, MON531, Kefeng 6, and pCambia-1381, had duplicated enhancer regions; these were defined as double enhancer promoters (Table 1).

Twenty-four different detection methods targeting the P35S promoter were identified from published papers and detection standards, and labeled as M1 to M24 (Table 2). Of these, the M1 method was adopted by ISO 21569^15^, the M13 method by ISO 21570^32^, three methods (M1, M14, and M11) by the National Standards of China^11^,^28^, four (M1, M2, M15, and M16) by the Industrial Standards of China^10^,^16^,^35^, and four (M1, M4, M12, and M14) were collected by the EU Database of Reference Methods for GMO Analysis (http://gmo-crl.jrc.ec.europa.eu/gmomethods/)^14^,^31^,^34^,^45^. These 24 methods included 10 conventional qualitative PCR methods and 14 real-time PCR methods. In addition, partial primer pairs for real-time PCR methods, such as M1, M3, M4, M5, M12, and M14, were simultaneously used for conventional qualitative detection (Table 2). Primer and probe sequences are given in Table 2. Primer pairs (or primer/probe sets) were aligned with the whole genome of CaMV using the bl2seq program, and their positions shown in Table 2. According to the positions of primers/probes in the CaMV genome, we observed that with the exception of five methods (M2, M7, M10, M12, and M18) the reverse primers of 19 methods were located outside of the conserved region of P35S. The P35S sequences from the CaMV genome, multiple transgenic events, and binary vectors with SNPs, together with primer/probe sets, were aligned using AlignX in the Vector NTI 9 software suite. Sequence alignment revealed that the vast majority of these 19 methods contained SNPs in the binding site of primer (SNPs were also shown in Table 2), and their reverse primers mismatched the P35S of MON88913 cotton, 98140 maize and the P35S regulating Pat gene of T25 maize (Supplementary Fig. S1a-f online). Both the forward and the reverse primers of method M10 located outside the conserved region, mismatching the P35S of GTS-40-3-2, MON863, MON810, MON89034, and MON1445; and the forward primer of M10 located outside the conserved region, mismatching the P35S of NK603, Bt11, MON88017, MON15985, MON88913, MON531, Kefeng6, KMD, OXY235 and pCambia-1381 (Supplementary Fig S1g online). The forward primer of M18 did not match the P35S of GTS-40-3-2, MON863, MON89034, and MON1445 (Supplementary Fig S1h online). Sequence alignment revealed that 21 of the 24 published P35S-based methods had defects, resulting in missed detection of partial transgenic events; the exceptions were M2, M7, and M12, where both primers and probes were located within the conserved region of P35S.

**Figure 1:**
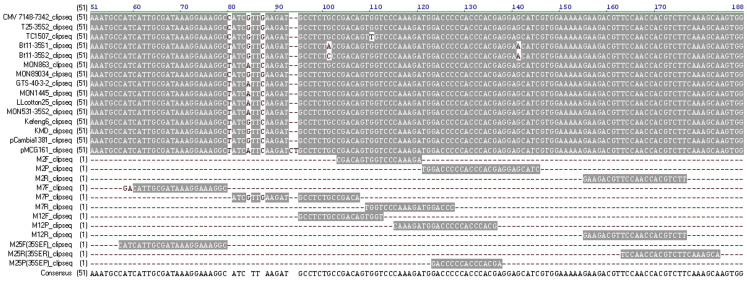
Sequence alignment of the P35S conserved region from CaMV, transgenic events and binary vectors harboring SNPs, together with primer/probe sets of the M2, M7, M12 methods and primer/probe set (M25) designed in this study.

To investigate the sequence consistency of primer binding sites for methods M2, M7, and M12, the P35S conserved region from the CaMV genome, multiple transgenic events, and binary vectors harboring nucleotide alterations, together with primer/probe sets, were aligned (Fig. 1). For the M2 method, a SNP in TC1507 maize located at the binding site of the forward primer, and a SNP in Bt 11 at the binding site of the probe. For the M7 method, the binding site of the probe was a high variability region containing four SNP mutations, resulting in a mismatch with most of the transgenic events and vectors. Furthermore, the SNP in TC1507 also existed in the binding site of the reverse primer, corresponding to the second nucleotide of the 3′ end of the reverse primer. For the M12 method, two SNPs in TC1507 and Bt11 were both situated in the binding site of the forward primer, with the SNP in TC1507 corresponding to the third nucleotide of the 3′ end of the primer. We speculated that the nucleotide mutation in the primer binding sites would cause inefficient amplification of methods M2, M7, and M12, and that the M7 probe located in a highly variable region could give rise to an abnormal fluorescent signal when detecting mutated P35S targets.

### Qualitative detection of P35S in GM crops using the collected methods

**Figure 2:**
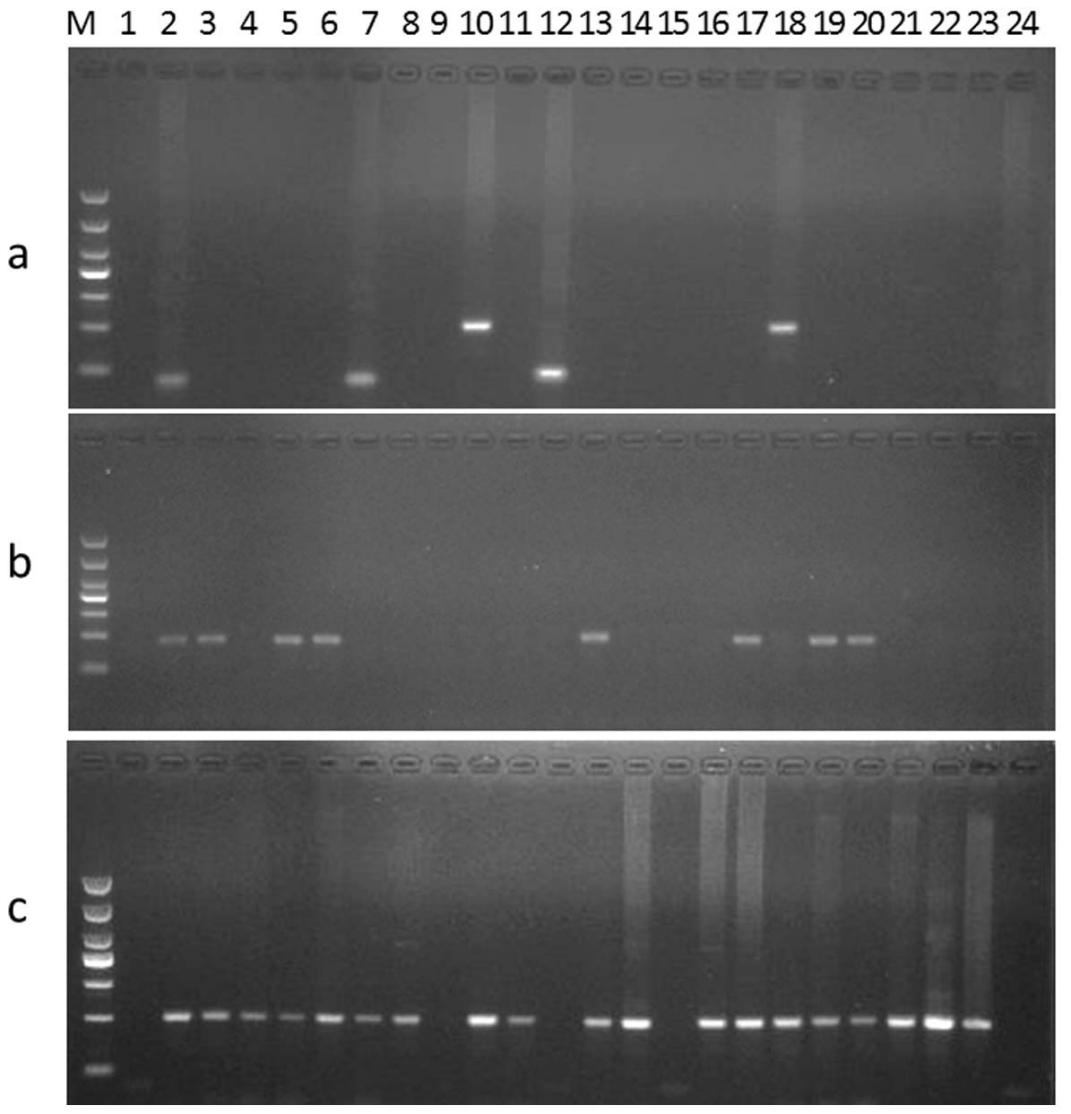
PCR amplification of P35S targets in GM crops using existing methods. (a) Amplification of the P35S fragment in MON 88913 event using the 24 existing P35S-based methods, Lanes 1–24 correspond to the M1-M24 methods. (b–c) Amplification of the P35S fragments in 23 transgenic events by methods M10 and M18, respectively. Lanes 1–24 correspond to the following samples: GM soybean (1) GTS40-3-2, (2) A5547-127, (3) A2704-12; GM maize (4) Bt11, (5) TC1507, (6) T25, (7) Bt176, (8) NK603, (9) MON89034, (10) M88017, (11) MON810, (12) MON863, (13) 59122; GM cotton (14) MON88913, (15) MON1445, (16) MON531, (17) LLcotton25, (18) MON15985; GM rapeseed (19) T45, (20) Topas19/2, (21) OXY235; GM rice (22) Kefeng 6, (23) KMD; (24) non-GM crop mixture (soybean, maize, cotton, rapeseed and rice).

The 24 collected P35S-based methods were used to detect P35S in MON88913. Since at least one primer lied outside the P35S region of MON88913, nineteen primer pairs failed to detect the P35S target when MON88913 genomic DNA was used as template; while five methods (M2, M7, M10,M12 and M18) successfully detected the P35S target (Fig. 2a). Sequence comparison indicated that the above 19 methods would also fail to detect P35S in 98140 maize, whereas, this was unable to be confirmed due to unavailable to 98140 maize (Supplementary Fig. S1a–f online). The M10 method was used to amplify P35S fragments from 23 GM varieties, including GM soybean GTS40-3-2, A5547-127, A2704-12; GM maize Bt11, TC1507, T25, Bt176, NK603, MON89034, M88017, MON810, MON863, 59122; GM cotton MON88913, MON1445, MON531, LLcotton25, MON15985; GM rapeseed T45, Topas19/2, OXY235; and GM rice Kefeng 6, and KMD. The 188-bp amplicon was not visualized in 15 of the samples: GTS40-3-2, Bt11, Bt176, NK603, MON89034, MON88017, MON810, MON863, MON88913, MON1445, MON531, MON15985, OXY235, Kefeng6, and KMD (Fig. 2b). The 23 GM varieties tested above were also analyzed for the existence of P35S by the M18 method. Of those, four GM crops (GTS-40-3-2, MON89034, MON863, and MON1445) were failed to yield an expected 196-bp PCR fragment (Fig. 2c). The expected product was observed in MON531 cotton when using the M18 method, because two copies of P35S are present in MON531, with one copy having completely matched primer binding sites for M18 (Supplementary Fig. S1h online). The detection results for P35S in GM crops were in agreement with the above sequence alignment results. The qualitative detection of P35S demonstrated that most existing P35S-based methods had flaws resulting in missed detection of partial GM crops harboring P35S.

### Influence of SNP mutations on PCR performance of the M2, M7 and M12 methods

**Figure 3:**
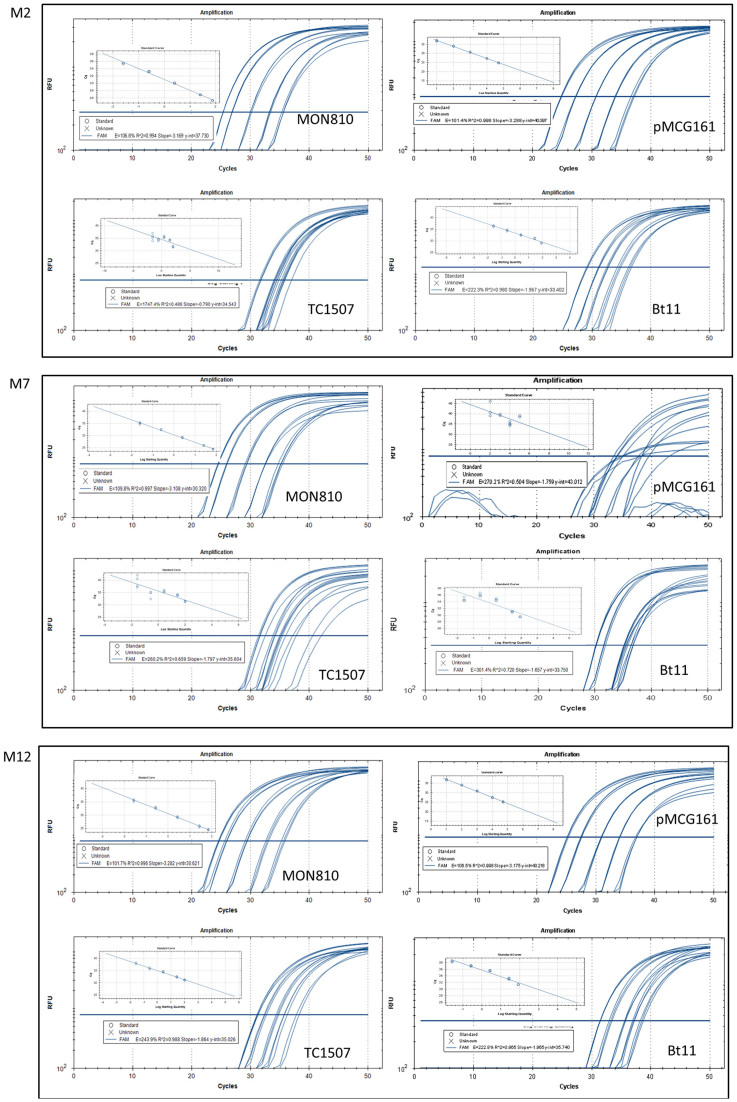
TaqMan assays using M2, M7, and M12 methods with serial DNA dilutions from events MON810, TC1507, Bt11, and the vector pMCG161. Standard curves were constructed based on the amplification plot. MON810 maize, which does not contain a SNP, was used as the control. Methods M2, M7 and M12 were assessed with events TC1507, Bt11, and vector pMCG161.

The binding sites of the primer/probe set contained SNPs for the M2, M7, and M12 methods (Fig. 1). To evaluate the effect of SNP mutations on the PCR performance of these three methods, a series of dilutions of extracted DNAs from MON810, TC1507, and Bt11 events and the binary vector pMCG161 were used as calibrators to set up standard curves, with each of the five dilutions assayed in triplicate. According to the sequence alignment results, the DNA from MON810 contained no SNPs, and could therefore be used as a control; DNA from events Bt11, TC 1507 and plasmid pMCG161 was used to assess the influence of the primer/probe mismatch in the M2, M7, and M12 methods; The amplification plots and corresponding standard curves are shown in Fig. 3, the R^2^ and slope data of the standard curves are summarized in Table 3, and the Ct values listed in Supplementary Table S2 online. The characteristic parameters of the standard curves constructed using MON810 were in the acceptable range for all three methods, with a slope range from −3.108 to −3.282, and R^2^ values ranging from 0.994 to 0.997^53^. The amplification plot of Bt11 showed obvious gradient changes among serial dilutions for the M2 and M12 methods, but the standard curve had a very shallow slope (−1.967 for M2, −1.965 for M12), exceeding the acceptable range (−3.1 to −3.6). No gradient change and poor repeatability among the three parallel reactions were visualized using Bt11 with the M7 method, resulting in standard curves with a poor correlation coefficient (0.72) and extreme slope (−1.657). The TaqMan assays for the M2 and M7 methods with TC1507 showed extreme slopes and abnormal amplification curves, similar to that seen when Bt11 was assayed by M7; for the M12 method, the TaqMan assays with TC1507 also exhibited an abnormal slope (−1.864), but obvious gradient changes among the serial dilutions and good repeatability among parallel reactions were observed. Partial dilutions of pMCG161 were assayed using the three methods, the characteristic parameters of the standard curves were in the acceptable range for methods M2 and M12, whereas, the M7 method generated poor fluorescent signals, and relatively large Ct values.

The TaqMan assays revealed that the SNP mutation in TC1507 generated large anomalies in the PCR performance of the M2, M7, and M12 methods, in agreement with the previous study^9^. The pMCG161 plasmid had two additional nucleotides and two SNP at the binding site of the M7 probe compared to the other events (Fig. 1), leading to very poor fluorescent amplification curves. For the Bt11 event, two SNP sites at the M7 probe binding site severely affected the PCR performance of the M7 method, one SNP at the M2 probe binding site and the M12 forward primer binding site only resulted in larger Ct values and a shallower slope. In conclusion, the mismatch between primer/probe and DNA template influenced the amplification plot and the characteristic parameters of the standard curves.

### Primer/probe design

A fragment of approximately 195 bp between position 7148 and 7342 in the CaMV genome was relatively conserved across different P35S promoters, even though it contained multiple SNPs dividing it into smaller discrete segments. This conserved fragment was present in all collected P35S sequences in this study, furthermore, two copies were contained in double enhancer promoters such as the enhanced P35S promoter in pCambia-1381, maize NK603 and MON88017, cotton MON15985, MON88913 and MON531, and rice Kefeng6. Multiple candidate primer/probe sets were designed to anneal to conserved segments carrying no SNP. To select the best primer/probe set, all possible primer and probe combinations were tested for amplicon size and specificity using 0.1 ng genomic DNA from transgenic maize TC1507 as a template, which possesses a P35S promoter and has been observed to have inefficient amplification in some of the methods. The most effective, reliable, and robust primer/probe set was 35SEF/35SER/35SEP, yielding a 125 bp amplicon, and labeled as M25 (Table 2). The binding sites of the selected primers/probe had 100% identity to all of the tested P35S promoter fragments (Fig. 1).

### Conventional PCR detection of the P35S promoter

**Figure 4:**
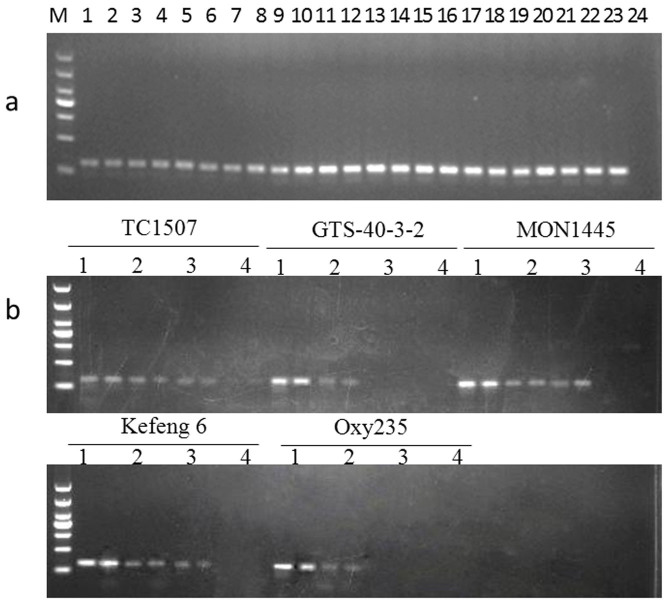
Testing of the amplification stability and sensitivity of the qualitative PCR detection. (a) Amplification of P35S in different GM crops. Key: Lane M, DL 1000 DNA Marker, Lanes 1–24 correspond to the following samples: GM soybean (1) GTS40-3-2, (2) A5547-127, (3) A2704-12; GM maize (4) Bt11, (5) TC1507, (6) T25, (7) Bt176, (8) NK603, (9) MON89034, (10) M88017, (11) MON810, (12) MON863, (13) 59122; GM cotton (14) MON88913, (15) MON1445, (16) MON531, (17) LLcotton25, (18) MON15985; GM rapeseed (19) T45, (20) Topas19/2, (21) OXY235; GM rice (22) Kefeng 6, (23) KMD; (24) non-GM crop mixture (soybean, maize, cotton, rapeseed and rice). (b) Sensitivity of the qualitative PCR method. Serially diluted DNA extracts of maize TC1507, soybean GTS 40-3-2, cotton MON 1445, rice Kefeng 6, and rapeseed OXY235 were used as templates. Lanes 1–4 correspond to 100, 50, 20, and 10 haploid genome copies, respectively; each template was run with two parallel PCR reactions.

The amplification stability of the primer pair 35SEF/35SER was tested using the genomic DNA from the 23 GMOs described above. Electrophoresis revealed that the unique 125 bp fragment was amplified from all samples containing a P35S promoter (Fig. 4a). Specificity testing revealed that no amplification occurred in samples lacking the promoter (data not shown). Therefore, conventional PCR amplification using the primer pair 35SEF/35SER reliably and specifically detected the P35S promoter in GM crops.

In practice, DNA extracted from GM food or feed tends to be highly degraded or very low in quantity. To evaluate the detection sensitivity of the new qualitative detection system, genomic DNA from five transformants (TC1507, GTS-40-3-2, MON1445, KMD, and OXY235) containing the P35S promoter, representing the five major transgenic crops (soybean, maize, rapeseed, rice, and cotton), were serially diluted to 100, 50, 20, and 10 copies per microliter and used as templates for PCR analysis. The lowest detectable template quantity required for maize TC1507 was estimated to be 20 copies; for soybean GTS40-3-2, 50 copies; for cotton MON1445, 20 copies; for rice KMD, 20 copies; and for rapeseed OXY235, 50 copies (Fig. 4b). These differences in PCR results may be because of different DNA organization. These results indicated that the detection sensitivity of our new qualitative method was as low as 50 copies or fewer in the tested species.

### Performance of P35S quantitative method on different GM crops

**Figure 5:**
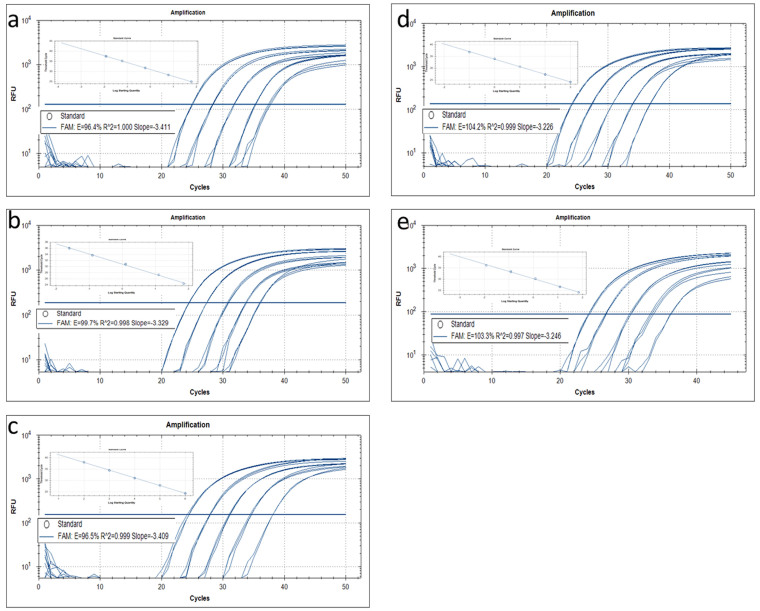
Amplification plot and standard curves for real-time quantitative PCR assays of P35S using serially diluted genomic DNA from five transgenic events as calibrators. (a–e) correspond to the amplification plots and standard curves of events GTS-40-3-2, MON1445, OXY235, KMD1, and TC1507, respectively.

Variations in the DNA templates of different crops may affect test results during GMO screening by P35S-targeted methods. To evaluate the suitability of the newly developed P35S-based quantitative method for detecting various GM crops, the five events (TC1507, GTS-40-3-2, MON1445, KMD, and OXY235) were subjected to real-time PCR to analyze the performance of the quantitative PCR. The conserved regions in GTS-40-3-2 soybean, TC1507 maize, KMD rice, and MON1445 cotton all have SNPs present, while OXY235 rapeseed does not. A series of dilutions for each of the extracted DNAs was made, corresponding to 50000, 5000, 500, 50, and 10 copies/µL for events GTS-40-3-2, OXY235, TC1507, and KMD, and to 28000, 5000, 500, 50, and 10 copies/µL for event MON1445. The serial dilutions were used as calibrators to establish standard curves for P35S detection, and each dilution was assayed in triplicate. Standard curves were created by plotting Ct values against the logarithm of transgene copy numbers, good agreement was observed between the quantity of template and the Ct values for each event (Fig. 5). The square regression coefficients (R^2^), slope, and amplification efficiency are summarized in Table 4; these meet the minimum performance requirements for analytical methods of GMO testing defined by the European Network of GMO Laboratories (ENGL)^53^. The R^2^ values ranged from 0.997 for TC1507 to 1.000 for GTS-40-3-2, this is significantly higher than the ENGL minimum requirement of 0.98. The slopes across the five events ranged from −3.411 in GTS-40-2 to −3.225 in KMD, this is within the acceptable range of −3.6 ≤ slope ≤ −3.1. Based on the slope of the standard curve, the efficiency of this P35S PCR method was estimated to be from 96.4% to 104.2%, close to the ideal efficiency of 100%. The real-time PCR assays verified that SNPs in conserved regions did not influence the amplification efficiency of the newly developed quantitative method. We therefore conclude that our real-time assay is suitable for quantifying the P35S promoter copy number in various GM crops.

To determine the limits of detection (LOD) and quantification (LOQ) of the real-time PCR method, five genomic DNA templates (TC1507, GTS-40-3-2, MON1445, KMD, and OXY235) from the five major crops were each diluted to 80, 50, 40, 20, 10, 5, and 1 copies/µL to perform real-time PCR assays in 10 replicates. The discrepancy in the Ct values across the ten replicates became larger with decreased template copy number (Table 5). All 10 PCR replicates had typical fluorescence amplification curves for the five tested GMOs when the template copy number increased to 10, whereas only partial reactions were positive when using five or one copy template dilutions. There were no visual differences in PCR performance across the different GM crops. Therefore, the LOD of our quantitative PCR method reached 10 copies for the different GM crops. The relative standard deviation (RSD) of Ct values among the 10 replicates did not exceed 25% with the reduction of template copy number; we speculate that the LOQ of our method was approximately 50 copies, based on the relationship between LOQ and LOD elucidated by the guidance document of the Joint Research Centre, Institute for Reference Materials and Measurements (JRC-IRMM)^54^.

## Discussion

Screening methods are often used directly to make a preliminary judgment on whether or not samples are GMOs. Precise and accurate detection methods are a prerequisite for reliable control of GMOs, and the screening method should be suitable for a wide range of GMOs. The survey on the GMO detection methods revealed that multiple qualitative or quantitative methods were developed and published for the same one target sequence, for instance, 24 methods targeting P35S were established, 14 methods targeting TNOS, 11 methods for *NPTII* gene, and 15 methods for *Bar* gene (http://gmdd.shgmo.org/). Method heterogeneity across testing laboratories can lead to adverse testing results during the screening phase of GMO detection, which can consequently cause problems for international trade. Due to the difficulty in detecting all the different transgene events, a single artificial sequence is recommended to be developed as a universal barcode, which would not be found in natural DNA sources. During the process of developing GMOs in future, all transgene developers could be required to include the single artificial sequence that would make detection of all transgene events easier by one method. However, the implementation of this idea needs to obtain approval and support of both transgene developers and regulators.

P35S are likely to remain an important component of GMO products at present and in the foreseeable future. Currently, 24 methods targeting different regions of the P35S sequence are reported and adopted by individual research and testing laboratories^10^,^11^,^12^,^13^,^14^,^15^,^16^,^17^,^18^,^19^,^20^,^21^,^22^,^23^,^24^,^25^,^26^,^27^,^28^,^29^,^30^,^31^,^32^,^33^,^34^,^35^,^36^,^37^,^38^,^39^,^40^,^41^,^42^,^43^,^44^,^45^.This experiment demonstrated that all of the existing P35S screening systems had flaws, and that these may generate false negative results or/and underestimate GMO content during GMO detection. In this study, a conserved section in the enhancer region of P35S was identified by sequence alignment, and a general PCR method targeting this region was established. The validation results demonstrated that we have developed an improved general P35S screening system suitable for available transformants, whereas, we are still unable to test the method with all of the available commercial transgenic events. Sequence comparison revealed that many P35S sequences from transgenic events are different from each other. Because we do not have access to all existing approved GMOs that carry P35S, and unapproved GM materials are even more difficult to access, some nucleotide alterations may still be undiscovered in the P35S conserved region. While isolating the P35S sequences, we found that our DNA sequence data for the P35S region from GTS-40-3-2, TC1507, Bt11, T25, and MON1445 were inconsistent with the sequence information released by the GMDD database or described in US patent documents (Table 1). Therefore, it is important that testing laboratory staff pay attention to the sequence alterations of P35S introduced in transgenic events, check the homology of primer/probe sequences and templates, and to know the applicability of each method to any given sample; this will avoid detection errors during GMO screening.

Most existing GMO labeling systems are based on transgenic content, but not all transformants have a corresponding event-specific detection method. If a real-time PCR method targeting general transgenic components could be used for GMO quantitation, then GMOs without an event-specific detection method can be quantified and labeled. However, the new detection methods still have problems that require addressing because the copy number of the detecting target is not the same for different single copy transformation events. In this analysis the detection target locates in the enhancer region, which is usually reused in the double enhancer promoter to enhance regulatory activity. For instance, the NK603, MON88017, MON15985, MON88913, MON531, and Kefeng 6 events all contain a double enhancer promoter. Furthermore, events such as Bt11, T25, and MON531 contain two copies of P35S in their transgene constructs, and for the MON531 event one copy is a double enhancer promoter. Hence, GMO content can be overestimated when using the P35S method for events harboring a double enhancer promoter or multiple copies of P35S. Therefore, when this new quantitative PCR method is used to estimate the exogenous gene copy number of samples, DNA extracted from the same sample should be used to construct the standard curve. In addition, if only P35S shows positive signal for test samples in practice, a subsequent experiment detecting other genomic sequence of CaMV, should be performed to rule out CaMV itself as contaminant in the plant DNA samples. In conclusion, the use of this new P35S method, which covers a wide range of GMOs, will lead to more consistent results of GMO detection during the screening phase among different testing laboratories.

## Methods

### Plant materials

Transgenic materials, including seed powder of transgenic soybean (10% GTS 40-3-2) and transgenic maize (10% 59122, 5% BT11, 5% BT176, 10% MON810, 10% MON863, 5% NK603, and 10% TC1507), were purchased from the Institute for Reference Materials and Measurements (IRMM, Geel, Belgium). Seed powder of transgenic maize (MON88017, MON89034), cotton (MON88913, MON1445, MON15985, and MON531), and leaf DNA of soybean (A5547-127), maize (T25), cotton (LLcotton25), and rapeseed (Topas 19/2, T45, and Oxy235) were purchased from the American Oil Chemists' Society (Champaign–Urbana, IL, USA). Transgenic soybean (A2704-12), transgenic rice (Kefeng 6, KMD) and non-transgenic crop seeds were already available in our own laboratory.

### DNA extraction

A DNeasy Plant Mini Kit (Qiagen, Hilden, Germany) was used to extract and purify genomic DNA from seeds or seed powders in accordance with manufacturer's instructions. DNA purity was checked using a NanoDrop 2000 spectrophotometer (Thermo Fisher, Waltham, MA, USA), and sample concentrations further quantified using a Versafluor Fluorometer (Bio-Rad, Hercules, CA, USA) with a Quanti-iT^TM^ PicoGreen® dsDNA Assay Kit (Invitrogen, Carlsbad, CA, USA).

### Cloning of the P35S Promoter

Primers used for isolating the P35S sequences from different transgenic events were designed using Primer Premier 5.0 software (PREMIER Biosoft International, Palo Alto, CA) according to the nucleotide sequences flanking the P35S promoter. Primers were synthesized by Sangon (Shanghai, China); their sequences are given in Supplementary Table S3 online. The PCR samples were prepared using a KOD-Plus kit (Toyobo, Osaka, Japan) in a sample volume of 50 μL containing 20 ng of genomic DNA, 1× KOD-Plus PCR buffer, 200 μM of each dNTP, 1 mM MgSO4, 100 nM of each primer, and 0.5 units of KOD-Plus DNA polymerase. PCRs were performed on a C1000™ Thermal Cycler (Bio-Rad, Hercules, USA) using the following program: 94°C for 2 min (initial denaturation); 35 cycles of 94°C for 15 s (denaturation) and 68°C for 3 min (annealing and extension); and 68°C for 7 min (final extension). PCR products harboring a target band were recovered and subcloned into the pZErO-2 vector (Invitrogen, Carlsbad, CA, USA) via an *Eco*RV restriction enzyme site. Ligation products were transformed into *Escherichia coli* strain TOP10F (Invitrogen, Carlsbad, CA, USA), and positive clones screened. Plasmids containing the PCR products were sequenced using M13 forward and reverse primers (Tsingke, Beijing, China).

### Sequence alignment

Pairwise comparison of nucleotide sequences was performed using the bl2seq program available from the National Center for Biotechnology Information (NCBI) (http://blast.ncbi.nlm.nih.gov/Blast.cgi?PAGE_TYPE=BlastSearch&BLAST_SPEC=blast2seq&LINK_LOC=align2seq). Regions conserved in aligned P35S promoters were discovered based on the positions of different P35S promoters in the CaMV genome. The P35S region across the GMOs and binary vectors, together with the whole genome sequence of CaMV (NC_001497.1), were aligned using the AlignX program of the Vector NTI 9 software suite (Invitrogen).

### Primers and probes

Oligonucleotide primers and TaqMan fluorescent dye-labeled probes were designed according to the conserved region of P35S promoters in the relevant GM crops using a specified optimal melting temperature of approximately 60°C for primers and 70°C for probes. The 5′ ends of probes were labeled with the fluorescent reporter 6-carboxy-fluorescein (FAM), and the 3′ ends with the minor groove binder non-fluorescent quencher (MGBNFQ). All primers and fluorescent probes were synthesized by Sangon Biotech (Shanghai, China).

### PCR reactions

Conventional PCR was run on a Bio-Rad C1000™ Thermal Cycler using an optimized conventional PCR mixture: 1× PCR buffer (with 1.5 mM MgCl_2_), 200 μM of each dNTP, 0.25 μM of each primer, 1 U EX Taq™ (TaKaRa, Otsu, Japan), and 20 ng genomic DNA in a total volume of 20 µL. The PCR used the following cycle conditions: initial denaturation for 120 s at 94°C; 35 cycles of 30 s at 94°C, 30 s at 60°C, and 30 s at 72°C; and terminal elongation for 2 min at 72°C. PCR products were size fractionated using 2% agarose gel electrophoresis in 1× TAE buffer, and visualized with ethidium bromide. The UV-fluorescent emission was recorded with a Gel Doc XR system using Quantity One software (Bio-Rad).

Real-time PCR assays were carried out on a CFX96 Real-Time System (Bio-Rad) in a final volume of 20 µL. The reaction mixture for the P35S promoter contained 1× TaqMan Universal PCR Master Mix (Applied Biosystems, Foster City, CA, USA), 400 nM primers, and 200 nM probe. All real-time PCRs were performed using the same program: pre-digestion at 50°C for 2 min; initial denaturation and uracil-N-glycosylase deactivation at 95°C for 10 min; and 50 cycles of 15 s at 94°C (denaturation) and 1 min at 60°C (annealing and extension). Fluorescence was measured after each annealing and extension step using CFX Manager ver. 1.6 (Bio-Rad). Data analysis was performed using the CFX Manager ver. 1.6 (1.6.541.1028) software (Bio-Rad).

## Author Contributions

G.W. designed this experiment and wrote the main manuscript; Y.W. designed this experiment, wrote the manuscript, and prepared figures; Y. Wang., J.L. and W.L. performed this experiment; L.Z. and Y.L. advised on the experimental design and commented on the manuscript; other authors, including X.L., L.Z. and J.L., reviewed the manuscript.

## Supplementary Material

Supplementary Information Development of a general method for detection and quantification of the P35S promoter based on assessment of existing methods

## Figures and Tables

**Table 1:** P35S sequence information from different transgenic events

					Identity of whole sequence		Identity of conserved region		
Crop	Transgenic event	Length	Accession No.	Homologous region with CaMV^a^	Percent	Number of SNPs	Percent	Number of SNPs	Note
Soybean	GTS-40-3-2	322	KJ608131	7148-7468	95%	16	93%	13	Different from GMDD^b^
	A5547-127	530	KJ608130	6908-7437	100%	0	100%	0	
Maize	MON863	322	KJ608136	7148-7468	95%	16	93%	13	
	NK603	551	KJ608140	7090-7344,7090-7381	100%	0	100%	0	Double enhancer
	TC1507	651	KJ608142	6913-7565	99%	6	99%	1	Different from GMDD^c^
	Bt11	502	KJ608143	7072-7563	95%	26	94%	11	Different from GMDD^d^
		501	KJ608144	7072-7563	95%	28	94%	11	
	MON810	307	KJ608135	7137-7443	100%	0	100%	0	
	T25	530	KJ608147	6908-7437	100%	0	99%	2	
		319	KJ608129	7027-7342	98%	5	100%	0	Different from GMDD^e^
	59122	530	US20060070139	6908-7437	100%	0	100%	0	
	MON88017	615	US8212113	7090-7344,7090-7443	100%	0	100%	0	Double enhancer
	MON89034	296	US8062840	7148-7443	97%	9	95%	9	
	Bt176	203		7365-7567	98%	4	\	\	Not complete
	98140	436	US7897846B2	6909-7344	100%	0	100%	0	
		438	US7897846B2	6909-7346	100%	0	100%	0	
		438	US7897846B2	6909-7346	100%	0	100%	0	
Cotton	MON15985	610	KJ608138	7090-7344,7090-7438	100%	0	100%	0	Double enhancer
	MON88913	519	KJ608139	7090-7347,7090-7344	100%	0	100%	0	Double enhancer
	MON531	615	KJ608145	7090-7344,7090-7443	100%	0	100%	0	Double enhancer
		322	KJ608146	7148-7468	95%	16	93%	13	
	LL25	1385	KJ608134	6105-7483	94%	78	95%	10	
	MON1445	322	KJ608137	7148-7468	95%	16	93%	13	Different from GMDD^f^
Rapeseed	Oxy235	840	KJ608141	6495-6972,7090-7443	99%	3	100%	0	
	T45	530		6908-7437	99%	1	100%	0	
	Topas 19/2	530		6908-7437	99%	1	100%	0	
Rice	Kefeng6	787	KJ608132	7017-7343,7018-7478	95%	32	94%	11	Double enhancer
	KMD	462	KJ608133	7017-7478	97%	16	94%	11	
	LLRice602	530	KF036176	6908-7437	99%	1	100%	0	
Binary	pBI121	835	AF485783	6605-7439	99%	3	100%	0	
vector	pCAMBIA1381	781	AF234302.1	7018-7445,7021-7345	96%	17	94%	11	Double enhancer
	pMCG161	1332	AY572837.1	6114-7440	96%	55	94%	12	

^a^the accession number of CaMV is NC_001497.1 in the GenBank database.

^b, c, d, e, f^ represent isolated sequences different from the released sequences in the GMDD database, and correspond to the following urls: http://gmdd.shgmo.org/sequence/view/1, http://gmdd.shgmo.org/sequence/view/10, http://gmdd.shgmo.org/sequence/view/39, http://gmdd.shgmo.org/sequence/view/17, and http://gmdd.shgmo.org/sequence/view/23.

**Table 2:** Primers and fluorescent probes used in the qualitative and real-time quantitative PCR systems to detect the P35S promoter

No.	Orientation	Name	Sequence(5′-3′)^b^,c	Position^a^	Amplicon	Reference	Note
M1	forward	35S-1	GC**T**CCTACAAATGCCATCA	7190–7208	195	10,11,12,13,14,15	
	reverse	35S-2	GATAGTGGGATTGTGCGTCA	7365–7384		10,11,12,13,14,15	
	probe	35S core	**TC**TCCACTGACGTAAGGGATGACGCA	7346–7371		13	
M2	forward	35S-F3	CGACAGT**G**GTCCCAAAGA	7248–7265	74	16	
	reverse	35S-R3	AAGACGTGGTTGGAACGTCTTC	7300–7321		16	
	probe	35S-P	TGGACCCCCACCCACGAGGA**G**CATC	7266–7290		16	
M3	forward	P35S 1-5′	ATTGATGTGA**T**A**TC**TCCACTGACGT	7334–7358	101	17,18,19,20	
	reverse	P35S 2-3′	CCTCTCCAAATGAAATGAACTTCCT	7410–7434		17,18,19,20	
	probe	P35S-Taq	CCC**A**CTATCCTTCGCAAGACC**C**TTCCT	7376–7402		18	
M4	forward	sF	CGTCTTCAAAGCAAGTGGAT**T**G	7316–7337	79	12,13,21,22	
	reverse	sR	TCTTGCGAAGGATAGTGGGATT	7373–7394		12,13,21,22	
	probe	P-35S_3-P	**TC**TCCACTGACGTAAGGGATGACGCA	7346–7371		13,22	
M5	forward	35SFZ1	CCGACAGT**G**GTCCCAAAGATGGAC	7247–7270	162	13,23	
	reverse	35SFZ2	ATATAGAGGAA**G**GGTCTTGCGAAGG	7384–7408		13,23	
	probe	35S core	**TC**TCCACTGACGTAAGGGATGACGCA	7346–7371		13	
M6	forward	P-35S_4-L	GACGTAAGGGATGACGCACAA	7354–7374	81	24	
	reverse	P-35S_4-R	CCTCTCCAAATGAAATGAACTTCCT	7410–7434		24	
	probe	P-35S_4-P	CCCACTAT**C**CTTCGCAAGACC**C**TTC	7376–7400		24	
M7	forward	35S-promoter.for	**GA**CATTGCGATAAAGGAAAGGC	7205–7226	68	25	
	reverse	35S-promoter.rev	GGGTCCATCTTTGGGA**C**CA	7254–7272		25	
	probe	35Spromoter-specific	ATC**G**TT**G**AAGATGCCTCT**G**CCGACA	7228–7252		25	
M8	forward	35SF	CCTACAAATGCCATCATTGCG	7193–7213	205	13	
	reverse	35SR	**G**GGTCTTGCGAAGGATAGTG	7378–7397		13	
	probe	35S Wolf	CAAAGATGGACCCCCACCCACG	7260–7281		13	
M9	forward	3-16f	CGTCTTCAAAGCAAGTGGAT	7316–7335	105	26	
	reverse	3-100r	GAA**G**GGTCTTGCGAAGGA	7383–7400		26	
	probe	3-67t	ACGCACAATCCCACTA	7367–7382		26	
M10	forward	P-35S_31-L	AGACTGGCGAACAGTTCATACAGA	6956–6979	188	27,28	Microarray
	reverse	P-35S_31-R	CAATGGAATCCGAGGAGGT	7124–7142		27,28	
	probe	P-35S_31-P	TG**C**TCCACCA**T**GTTGACGAAG	7011–7031		27,28	
M11	forward	P-35S-AF	AAGATGCCTCTGCCGACAGT	7235–7254	142	29	Microarray
	reverse	P-35S-AR	GATTGTGCGTCATCCCTTAC	7357–7376		29	
	probe	P-35S	GAACGTCTTCTTTTTCCACGATGCTCCTCG	7280–7309		29	
M12	forward	P180-F(TM-35S-F^33^,35S-FTM^31^)	GCCTCTGCCGACAGT**G**GT	7240–7257	82	12,30,31,32,33	
	reverse	P180-R(TM-35S-R^33^,35S-RTM^31^)	AAGACGTGGTTGGAACGTCTTC	7300–7321		12,30,31,32,33	
	probe	P180-P(TM-35S-Pro^33^,35S-TMP^31^)	CAAAGATGGACCCCCACCCACG	7260–7281		30,31,32,33	
M13	forward	P-35S-F	GACGTAAGGGATGACGCACAA	7354–7374	81	24,32	
	reverse	P-35S-R	**C**CTCTCCAAATGAAATGAACTTCCT	7410–7434		24,32	
	probe	P-35S-P	CCC**A**CTATCCTTCGCAAGACCCTTCC	7376–7401		24,32	
M14	forward	p35S-cf3	CCACGTCTTCAAAGCAAGTGG	7313–7333	123	11,12,13,34	
	reverse	p35S-cr4	TCCTCTCCAAATGAAATGAACTTCC	7411–7435		11,12,13,34	
	probe	35S core	**TC**TCCACTGACGTAAGGGATGACGCA	7346–7371		13	
M15	forward	35S-F	GC**T**CCTACAAATGCCATCATTGC	7190–7212	195	16,35	
	reverse	35S-R	GATAGTGGGATTGTGCGTCATCCC	7361–7384		16,35	
M16	forward	35S-F2	TCATCCCTTACGTCAGTGGA**G**	7347–7367	165	10	
	reverse	35S-R2	CCATCATTGCGATAAAGGAAA	7203–7223		10	
M17	forward	35SFZMP1(U-35S)	CCGACAGT**G**GTCCCAAAGATG	7247–7267	158	36,37	
	reverse	35SFZMP2(D-35S)	AGAGGAA**G**GGTCTTGCGAAGG	7384–7404		36,37	
M18	forward	SP1 F	TTGCTTTGAAGACGTGGTTG	7310–7329	196	38	
	reverse	SP1 R	ATTCCATTGCCCAGCTA**T**CT	7134–7153		38	
M19	forward	35S3F	GCCATCATTGCGATAAAGGAAAGG	7202–7225	173	39	
	reverse	35S6R	TTGTGCGTCATCCCTTACGTCAGTG	7350–7374		39	
M20	forward	CM01	**CA**CTACAAATGCCATCATTGCGATA	7192–7216	220	40	
	reverse	CM02	CTTATATAGAGGAA**G**GGTCTTGCGA	7387–7411		40	
M21	forward	35S-A	AA**G**GGTCTTGCGAAGGATAG	7380–7399	227	41	
	reverse	35S-B	AGT**G**GAAAAGGAAGGTGGC**T**	7173–7192		41	
M22	forward	35S F(p35S- ar1)	CCTACAAATGCCATCATTGCG	7193–7213	207	42,43	
	reverse	35S R (p35S-af1u)	**G**GGTCTTGCGAAGGATAGTG	7378–7397		42,43	
M23	forward	35S-111 F	GT**G**GTCCCAAAGATGGACCC	7253–7272	111	44	
	reverse	35S-111 R	CCCTTACGTCAGTGGAGATATC**A**CA	7339–7363		44	
M24	forward	35SA	AA**G**GGTCTTGCGAAGGATAG	7380–7399	149	42	Nested PCR
	reverse	35SB	AGT**G**GAAAAGGAAGGTGGC**T**	7173–7192		42	
	reverse	35SC	ACAGT**G**GTCCCAAAGATGGA	7250–7269		42	
M25	forward	35SEF	CATCATTGCGATAAAGGAAAGGC	7204–7226	125	This study	
	reverse	35SER	TGCTTTGAAGACGTGGTTGGA	7308–7328			
	probe	35SEP	TCGTGGGTGGGGGTC	7247–7268			

^a^Primer and probe positions are indicated relative to the genome sequence of CaMV (GenBank accession NC_001497.1).

^b^Probes of M1–M24 were labeled with 5′ FAM and 3′ BHQ1. The probe of M25 was labeled with 5′ FAM and 3′ MGBNFQ.

^c^Nucleotides mismatching the reference CaMV sequence or partial GMO templates, were highlighted in bold.

**Table 3:** Parameters of standard curves for the M2, M7, and M12 methods

Event	Parameter	M2	M7	M12
MON810	R^2^ value	0.994	0.997	0.996
	Slope	−3.169	−3.108	−3.282
TC1507	R^2^ value	0.486	0.659	0.988
	Slope	−0.790	−1.797	−1.864
Bt11	R^2^ value	0.980	0.720	0.965
	Slope	−1.967	−1.657	−1.965
pMCG161	R^2^ value	0.998	0.504	0.998
	Slope	−3.288	−1.759	−3.175

**Table 4:** Parameters of the P35S standard curves when using serially diluted genomic DNA from five events as calibrators

Events	R^2^ value	Slope	Amplification efficiency
GTS-40-3-2	1.000	−3.411	96.40%
TC1507	0.997	−3.246	103.30%
MON1445	0.998	−3.329	99.70%
OXY235	0.999	−3.409	96.50%
KMD	0.999	−3.226	104.20%

**Table 5:** Estimation of LOD and LOQ of real-time PCR methods when using serially diluted genomic DNA from five events as templates

Transgenic event	template copy no.	signal ratio	mean Ct value	SD	RSD
GTS-40-3-2	80	10/10	34.63	0.46	1.32
	50	10/10	35.11	0.31	0.89
	40	10/10	35.51	0.47	1.33
	20	10/10	36.28	0.31	0.86
	10	10/10	37.33	0.49	1.31
	5	8/10	\	\	\
	1	4/12	\	\	\
MON1445	80	10/10	33.27	0.24	0.73
	50	10/10	34.47	0.26	0.75
	40	10/10	34.96	0.27	0.78
	20	10/10	35.69	0.48	1.33
	10	10/10	36.64	0.49	1.32
	5	9/10	\	\	\
	1	4/12	\	\	\
OXY235	80	10/10	33.34	0.26	0.79
	50	10/10	34.47	0.26	0.75
	40	10/10	35.16	0.47	1.33
	20	10/10	36.80	0.39	1.07
	10	10/10	38.29	0.58	1.52
	5	2/10	\	\	\
	1	2/10	\	\	\
KMD	80	10/10	33.28	0.65	1.94
	50	10/10	34.40	0.37	1.08
	40	10/10	35.09	0.32	0.93
	20	10/10	36.50	0.49	1.34
	10	10/10	37.15	0.80	2.14
	5	3/10	\	\	\
	1	5/10	\	\	\
TC1507	80	10/10	32.40	0.38	1.18
	50	10/10	33.31	0.36	1.08
	40	10/10	34.03	0.44	1.30
	20	10/10	35.73	0.40	1.13
	10	10/10	36.17	0.57	1.59
	5	2/10	\	\	\
	1	5/12	\	\	\
