# Supplementary material for: Development of a general method for detection and quantification of the P35S promoter based on assessment of existing methods
Source: Sci Rep. 2014 Dec 8;4:7358. doi: 10.1038/srep07358 (PMC4258656; doi:10.1038/srep07358)
Supplement: Supplementary Information — Development of a general method for detection and quantification of the P35S promoter based on assessment of existing methods [file srep07358-s1.pdf]

# Development of a general method for detection and quantification of the P35S promoter based on assessment of existing methods

Yuhua Wu[#1,3], Yulei Wang[#1,2], Jun Li[1,3], Wei Li[1,3], Li Zhang[4], Yunjing Li[1,3], Xiaofei Li[1,3], Jun Li[1,3], Li Zhu[1,3], Gang Wu[1,3*]

[1]Key Laboratory of Oil Crop Biology of the Ministry of Agriculture, Oil Crops Research Institute, Chinese Academy of Agricultural Sciences, No. 2 Xudong 2nd Road, Wuhan 430062, People's Republic of China

[2] College of Life Sciences, Hubei University, No. 368 Friendship Avenue, Wuhan 430062, People's Republic of China

[3]Supervision and Test Center (Wuhan) for Environmental Safety of Genetically Modified Plants, Ministry of Agriculture, No. 2 Xudong 2nd Road, Wuhan 430062, People's Republic of China

[4]School of Life Science, South-Central University for Nationalities, Min-Yuan Road 708, Wuhan 430074, People's Republic of China

The supplementary information includes three tables and one figure. Table S1 provides the presence of P35S in GM crops collected in Agbios GM database. Table S2 summarizes the Ct values of TaqMan assays by methods M2, M7 and M12, with serial DNA dilutions from events MON810, TC1507, Bt11 and binary vector pMCG161 as templates. Table S3 provides the primer information used for isolating

P35S sequences from transgenic events. Figure 1S shows the alignment of P35S sequences with primer/probe sets.

Table S1 Statistics of presence of P35S in GM crops according to the data of Agbios GM database (http://cera-gmc.org/index.php?action=gm_crop_database)

| Crop | Event number | P35S | TNOS | Both or either |
|---|---|---|---|---|
| Soybean | 11 | 6 | 3 | 6 |
| Maize | 28 | 23 | 17 | 27 |
| Cotton | 12 | 7 | 7 | 9 |
| Argentine canola | 16 | 7 | 9 | 14 |
| Rice* | 5 | 4 | 3 | 5 |
| Wheat | 1 | 1 | 1 | 1 |
| Flax | 1 | 0 | 0 | 0 |
| Papaya | 2 | 2 | 2 | 2 |
| Plum | 1 | 1 | 1 | 1 |
| Alfalfa | 1 | 0 | 0 | 0 |
| Potato | 6 | 5 | 6 | 6 |
| Sugar beet | 3 | 2 | 1 | 2 |
| Tabbaco | 2 | 0 | 0 | 0 |
| Tomato | 6 | 5 | 3 | 5 |
| Squash | 2 | 2 | 0 | 2 |
| Melon | 1 | 0 | 0 | 0 |
| Chicory | 1 | 0 | 1 | 1 |
| Carnation | 1 | 1 | 1 | 1 |
| Polish canola | 2 | 1 | 0 | 1 |
| Total | 102 | 67 | 55 | 83 |

**Note: *indicates five rice events contain 2 events (LL RICR 62 and LL RICR 601) from Agbios GM database and three events (TT51-1, Kefeng6 and KMD) from papers.**

**Table S2 Ct values of serial DNA dilutions of MON810, TC1507, Bt11 and pMCG161 assayed by methods M2, M7 and M12.**

| Method | Event | copy number | Ct value 1 | Ct value 2 | Ct value 3 | Mean | SD | RSD (%) |
|---|---|---|---|---|---|---|---|---|
| M2 | MON810 | 27600 | 25.30 | 25.33 | 25.33 | 25.32 | 0.02 | 0.07 |
| | | 10000 | 26.85 | 26.84 | 26.91 | 26.87 | 0.04 | 0.14 |
| | | 1000 | 30.18 | 30.00 | 30.00 | 30.06 | 0.10 | 0.35 |
| | | 100 | 33.25 | 33.37 | 33.15 | 33.26 | 0.11 | 0.33 |
| | | 10 | 35.57 | 35.53 | 35.27 | 35.46 | 0.16 | 0.46 |
| | TC1507 | 37000 | 31.76 | 31.49 | 31.68 | 31.64 | 0.14 | 0.44 |
| | | 10000 | 34.20 | 34.44 | 34.22 | 34.29 | 0.13 | 0.39 |
| | | 1000 | 35.82 | 35.57 | 35.21 | 35.53 | 0.31 | 0.86 |
| | | 100 | 34.00 | 34.78 | 34.48 | 34.42 | 0.39 | 1.14 |
| | | 10 | 35.43 | 35.71 | 36.98 | 36.04 | 0.83 | 2.29 |
| | Bt11 | 31000 | 29.16 | 29.04 | 29.19 | 29.13 | 0.08 | 0.27 |
| | | 10000 | 31.19 | 31.04 | 31.14 | 31.12 | 0.08 | 0.25 |
| | | 1000 | 32.71 | 32.41 | 32.60 | 32.57 | 0.15 | 0.47 |
| | | 100 | 34.23 | 34.83 | 34.58 | 34.55 | 0.30 | 0.87 |
| | | 10 | 36.15 | 36.37 | 36.63 | 36.38 | 0.24 | 0.66 |
| | pMCG161 | 50000 | 24.89 | 24.74 | 24.83 | 24.82 | 0.08 | 0.30 |
| | | 10000 | 27.28 | 27.20 | 27.27 | 27.25 | 0.04 | 0.16 |
| | | 1000 | 30.71 | 30.69 | 30.64 | 30.68 | 0.04 | 0.12 |
| | | 100 | 34.02 | 33.91 | 34.03 | 33.99 | 0.07 | 0.20 |
| | | 10 | 37.01 | 36.58 | 37.17 | 36.92 | 0.31 | 0.83 |
| M7 | MON810 | 27600 | 24.40 | 24.52 | 24.42 | 24.45 | 0.06 | 0.26 |
| | | 10000 | 26.02 | 26.02 | 25.87 | 25.97 | 0.09 | 0.33 |
| | | 1000 | 29.36 | 29.25 | 29.06 | 29.22 | 0.15 | 0.52 |
| | | 100 | 32.44 | 32.47 | 32.53 | 32.48 | 0.05 | 0.14 |
| | | 10 | 35.14 | 35.41 | 35.20 | 35.25 | 0.14 | 0.40 |
| | TC1507 | 37000 | 31.59 | 31.52 | 31.32 | 31.48 | 0.14 | 0.45 |
| | | 10000 | 33.78 | 34.18 | 34.06 | 34.01 | 0.21 | 0.60 |
| | | 1000 | 35.40 | 36.00 | 35.21 | 35.54 | 0.41 | 1.16 |
| | | 100 | 34.96 | 35.11 | / | 35.04 | 0.11 | 0.30 |
| | | 10 | 37.75 | 37.18 | 40.56 | 38.50 | 1.81 | 4.70 |
| | Bt11 | 31000 | 29.51 | 29.56 | 29.48 | 29.52 | 0.04 | 0.14 |
| | | 10000 | 30.99 | 31.20 | 30.87 | 31.02 | 0.17 | 0.54 |
| | | 1000 | 34.76 | 35.00 | 34.36 | 34.71 | 0.32 | 0.93 |
| | | 100 | 35.63 | 35.95 | 36.05 | 35.88 | 0.22 | 0.61 |
| | | 10 | 35.48 | 34.35 | 34.57 | 34.80 | 0.60 | 1.72 |
| | pMCG161 | 50000 | / | 38.44 | 39.07 | 38.76 | 0.45 | 1.15 |
| | | 10000 | 34.3 | 34.91 | 35.06 | 34.76 | 0.40 | 1.16 |
| | | 1000 | 36.13 | 39.84 | 39.1 | 38.36 | 1.96 | 5.12 |

| | | | | | | | | |
|---|---|---|---|---|---|---|---|---|
| | | 100 | 39.01 | 40.58 | / | 39.80 | 1.11 | 2.79 |
| | | 10 | / | / | / | | | |
| M12 | MON810 | 27600 | 24.65 | 24.48 | 24.55 | 24.56 | 0.09 | 0.35 |
| | | 10000 | 25.99 | 25.56 | 25.88 | 25.81 | 0.22 | 0.87 |
| | | 1000 | 29.40 | 29.06 | 29.31 | 29.26 | 0.18 | 0.60 |
| | | 100 | 33.06 | 32.83 | 32.54 | 32.81 | 0.26 | 0.79 |
| | | 10 | 35.82 | 35.57 | 35.21 | 35.53 | 0.31 | 0.86 |
| | TC1507 | 37000 | 31.08 | 31.26 | 31.21 | 31.18 | 0.09 | 0.30 |
| | | 10000 | 32.66 | 32.28 | 32.41 | 32.45 | 0.19 | 0.60 |
| | | 1000 | 34.30 | 34.72 | 34.52 | 34.51 | 0.21 | 0.61 |
| | | 100 | 35.69 | 36.20 | 35.66 | 35.85 | 0.30 | 0.85 |
| | | 10 | 37.75 | 38.22 | / | 37.99 | 0.33 | 0.87 |
| | Bt11 | 31000 | 30.41 | 30.49 | 30.46 | 30.45 | 0.04 | 0.13 |
| | | 10000 | 32.01 | 32.44 | 32.27 | 32.24 | 0.22 | 0.67 |
| | | 1000 | 34.57 | 34.77 | 34.59 | 34.64 | 0.11 | 0.32 |
| | | 100 | 36.00 | 36.26 | 36.12 | 36.13 | 0.13 | 0.36 |
| | | 10 | 37.72 | 37.19 | 37.50 | 37.47 | 0.27 | 0.71 |
| | pMCG161 | 50000 | 25.10 | 25.14 | 25.29 | 25.18 | 0.10 | 0.40 |
| | | 10000 | 27.45 | 27.55 | 27.37 | 27.46 | 0.09 | 0.33 |
| | | 1000 | 30.86 | 31.00 | 30.94 | 30.93 | 0.07 | 0.23 |
| | | 100 | 33.99 | 34.06 | 34.00 | 34.02 | 0.04 | 0.11 |
| | | 10 | 36.91 | 36.92 | 36.67 | 36.83 | 0.14 | 0.38 |

**Table S3 Primers designed for isolating the P35S sequences from transgenic events**

| Crop | Transgenic event | primer name | Primer seqeunce | specificity |
|---|---|---|---|---|
| Soybean | GTS-40-3-2 | SGF | CCCTTCAATTTAACCGATGC | soybean genome |
| | | CTP4R | TAGCCACTGATGCTGAAATCCT | CTP4/antisense |
| | A5547-127 | SHA003F | GCTATTTGGTGGCATTTTTCCA | soybean genome |
| | | PatR | CAACACCCTCAACCTCAGCA | Pat/antisense |
| Maize | MON863 | PrimeF | TGTTACGGCCTAAATGCTGAAC T | Maize genome |
| | | NPTⅡR | AGCA GCCGATTGTCTGTTGTG | NPTII/antisense |
| | NK603 | TNOSF | ATCGTTCAAACATTTGGCA | NOS ter/ sense |
| | | Hsp70R1 | GATTGTGCGTCATCCCTTA | i-ZmHsp70/antisense |
| | TC1507 | 1507-3F | TCCACCAAGATGGAACTGC | ORF25/sense |
| | | PATR | CCAACCTTTGATGCCTATGTG | Pat/antisense |
| | Bt11 | PmF1 | TGAGCGAGGAAGCGGAAGA | Plasmid/sense |
| | | EnhR1 | GCGGCTTGTTGTGGTCTTTT | Adh1-enhancer/antisense |
| | | PmF2 | TGGCGTAATAGCGAAGAGGC | Plasmid/sense |
| | | EnhR2 | CCTTGGCCTCCCAGAAGTA | Adh1-enhancer/antisense |
| | MON810 | MGF | TCGAAGGACGAAGGACTCTAAC G | Maize genome |
| | | Hsp70R2 | TGCCCTATAACACCAACATGTG CTT | Hsp70 intron/antisense |
| | T25 | MGF1 | CAGCGACAATGGCGGAACGAC TCAA | Maize genome |
| | | LacZR | CACGCTGTAGGTATCTCAGTTC G | LacZ/antisense |
| | | LacZF | GTGAGCGAGGAAGCGGAAGA | lacZ/sense |
| | | PatR | CTGGCCTAATCTCAACTGGTCTC | Pat/antisense |
| Cotton | MON15985 | TNOS1F | TCGTTCAAACATTTGGCAATAA | NOS ter/ sense |
| | | Cry2AbR | TGAACGGCGATGCACCAATGTC | Cry2Ab/antisense |
| | MON88913 | TE9F | GATATGGTCCTTTTGTTCATTCT CA | T-E9 antisense |
| | | Actin8R1 | TACCATTGTCACAGACAATAGG TT | Act8 promoter from A. thaliana/antisense |
| | MON531 | CGF | GGCCAATGCCTCGTGAT | Cotton genome |
| | | NPTⅡR | CGGACAGGTCGGTCTTGACA | NPTII/antisense |
| | | Cry1AcR | ACGGAGGCATAGTCAGCAGGAC C | Cry1Ac/antisense |
| | | aadR | CCGGCAGGCGCTCCATTG | aad/antisense |
| | LL25 | KVM156F | CAAGGAACTATTCAACTGAG | Cotton genome |
| | | BarR | GAGGTCGTCCGTCCACTC | Bar/antisense |
| | MON1445 | OriVR | AAGGGTTTCCGCCCGTTT | OriV |
| | | NPTⅡR: | CGGACAGGTCGGTCTTGACA | NPTII/antisense |

| | | | | |
|---|---|---|---|---|
| Rapeseed | Oxy235 | RGF | CTTTACGGCGAGTTCTGTTAGG T | Rapeseed genome |
| | | OXY235R | GTTCGCTGTACCCAAAGGAGA | Bxn/antisense |
| Rice | Kefeng6 | Cry1AcF | CAGCATTCGTGAGGCTTACTTG | Cry1Ac/sense |
| | | HptR | GGCGAAGAATCTCGTGCTTTC | Hpt/antisense |
| | KMD | TNOS-2F | CAAAATATAGCGCGCAAA | NOS ter/sense |
| | | HptR | CCGCTCGTCTGGCTAAGATC | Hpt/antisense |

**Figure S1**

a

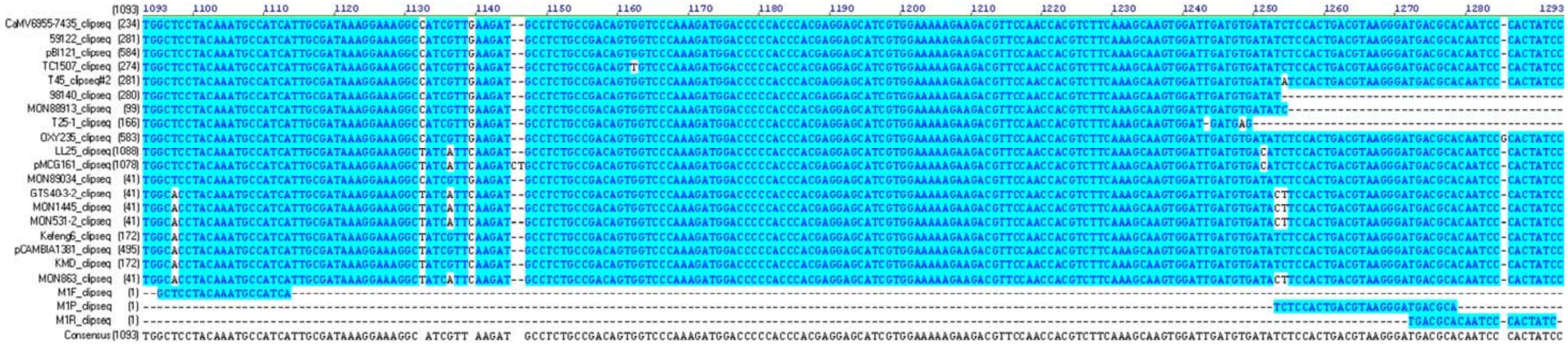

b

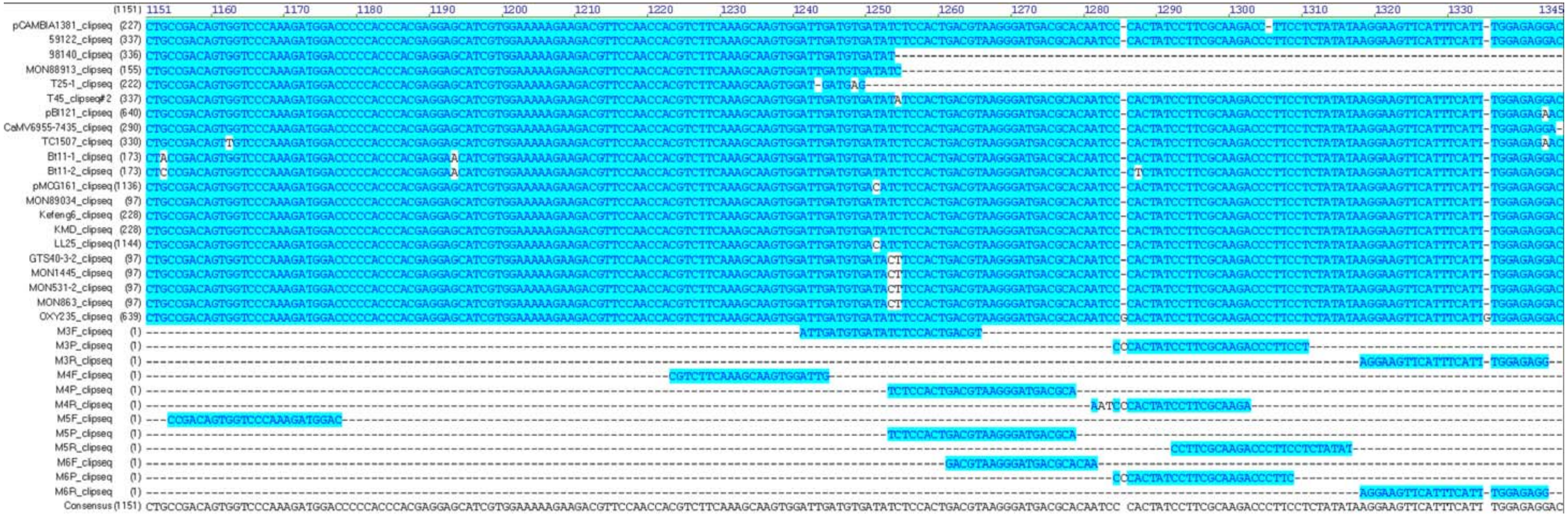
(1151) 1151 1160 1170 1180 1190 1200 1210 1220 1230 1240 1250 1260 1270 1280 1290 1300 1310 1320 1330 1345
pCAMBIA1381_clipseq (227)
59122_clipseq (337)
98140_clipseq (336)
MON88913_clipseq (155)
T25-1_clipseq (222)
T45_clipseq#2 (337)
pBI121_clipseq (640)
CaMV6955-7435_clipseq (290)
TC1507_clipseq (330)
Bt11-1_clipseq (173)
Bt11-2_clipseq (173)
pMCG161_clipseq (1136)
MON89034_clipseq (97)
Kefeng6_clipseq (228)
KMD_clipseq (228)
LL25_clipseq (1144)
GTS40-3-2_clipseq (97)
MON1445_clipseq (97)
MON531-2_clipseq (97)
MON863_clipseq (97)
OXY235_clipseq (639)
M3F_clipseq (1)
M3P_clipseq (1)
M3R_clipseq (1)
M4F_clipseq (1)
M4P_clipseq (1)
M4R_clipseq (1)
M5F_clipseq (1)
M5P_clipseq (1)
M5R_clipseq (1)
M6F_clipseq (1)
M6P_clipseq (1)
M6R_clipseq (1)
Consensus (1151)

C

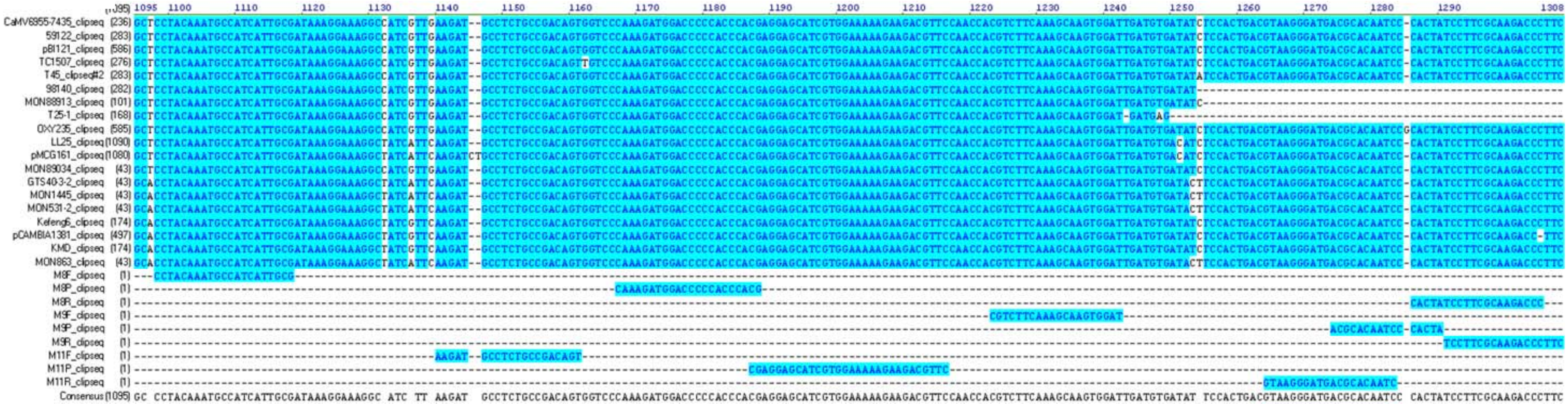

d

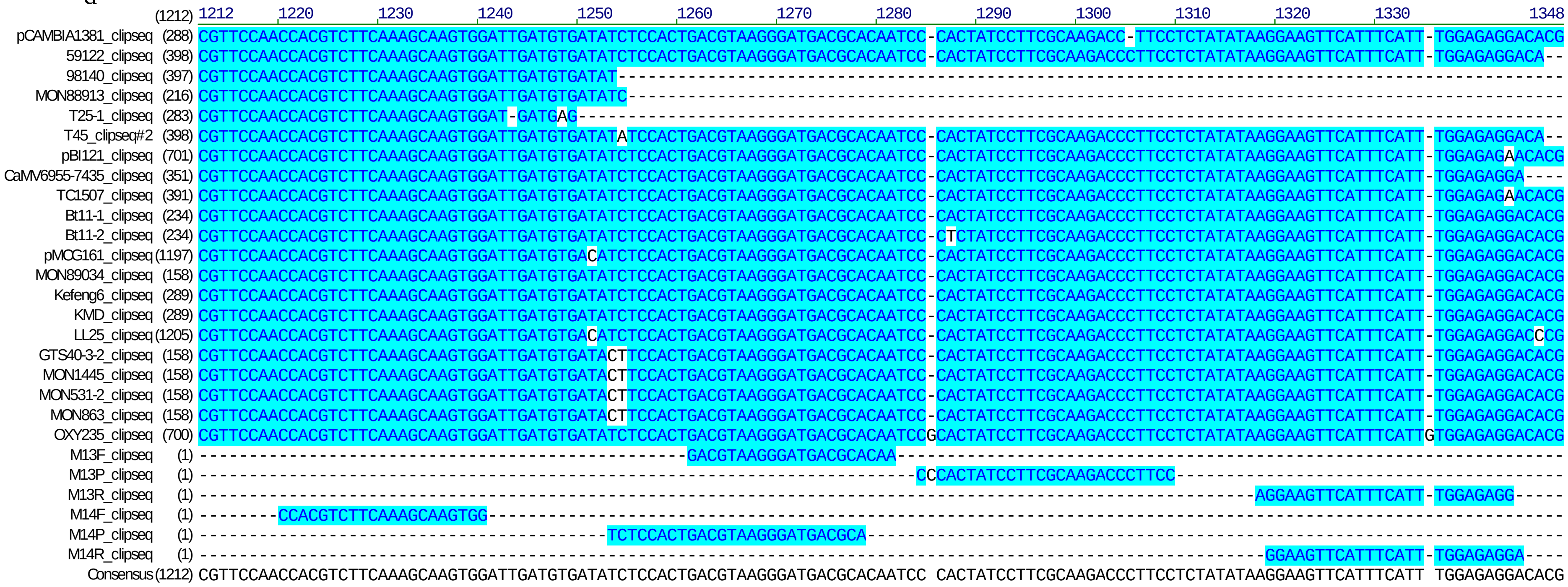
(1212) 1212 1220 1230 1240 1250 1260 1270 1280 1290 1300 1310 1320 1330 1348
pCAMBIA1381_clipseq (288) CGTTCCAACCACGTCTTCAAAGCAAGTGGATTGATGTGATATCTCCACTGACGTAAGGGATGACGCACAATCC-CACTATCCTTCGCAAGACC-TTCCTCTATATAAGGAAGTTCATTTCATT-TGGAGAGGACACG
59122_clipseq (398) CGTTCCAACCACGTCTTCAAAGCAAGTGGATTGATGTGATATCTCCACTGACGTAAGGGATGACGCACAATCC-CACTATCCTTCGCAAGACCCTTCCTCTATATAAGGAAGTTCATTTCATT-TGGAGAGGACA--
98140_clipseq (397) CGTTCCAACCACGTCTTCAAAGCAAGTGGATTGATGTGATAT-----------------------------------------------------------------------------------------------
MON88913_clipseq (216) CGTTCCAACCACGTCTTCAAAGCAAGTGGATTGATGTGATATC----------------------------------------------------------------------------------------------
T25-1_clipseq (283) CGTTCCAACCACGTCTTCAAAGCAAGTGGAT-GATGAG---------------------------------------------------------------------------------------------------
T45_clipseq#2 (398) CGTTCCAACCACGTCTTCAAAGCAAGTGGATTGATGTGATATATCCACTGACGTAAGGGATGACGCACAATCC-CACTATCCTTCGCAAGACCCTTCCTCTATATAAGGAAGTTCATTTCATT-TGGAGAGGACA--
pBI121_clipseq (701) CGTTCCAACCACGTCTTCAAAGCAAGTGGATTGATGTGATATCTCCACTGACGTAAGGGATGACGCACAATCC-CACTATCCTTCGCAAGACCCTTCCTCTATATAAGGAAGTTCATTTCATT-TGGAGAGAACACG
CaMV6955-7435_clipseq (351) CGTTCCAACCACGTCTTCAAAGCAAGTGGATTGATGTGATATCTCCACTGACGTAAGGGATGACGCACAATCC-CACTATCCTTCGCAAGACCCTTCCTCTATATAAGGAAGTTCATTTCATT-TGGAGAGGA----
TC1507_clipseq (391) CGTTCCAACCACGTCTTCAAAGCAAGTGGATTGATGTGATATCTCCACTGACGTAAGGGATGACGCACAATCC-CACTATCCTTCGCAAGACCCTTCCTCTATATAAGGAAGTTCATTTCATT-TGGAGAGAACACG
Bt11-1_clipseq (234) CGTTCCAACCACGTCTTCAAAGCAAGTGGATTGATGTGATATCTCCACTGACGTAAGGGATGACGCACAATCC-CACTATCCTTCGCAAGACCCTTCCTCTATATAAGGAAGTTCATTTCATT-TGGAGAGGACACG
Bt11-2_clipseq (234) CGTTCCAACCACGTCTTCAAAGCAAGTGGATTGATGTGATATCTCCACTGACGTAAGGGATGACGCACAATCC-CTCTATCCTTCGCAAGACCCTTCCTCTATATAAGGAAGTTCATTTCATT-TGGAGAGGACACG
pMCG161_clipseq (1197) CGTTCCAACCACGTCTTCAAAGCAAGTGGATTGATGTGACATCTCCACTGACGTAAGGGATGACGCACAATCC-CACTATCCTTCGCAAGACCCTTCCTCTATATAAGGAAGTTCATTTCATT-TGGAGAGGACACG
MON89034_clipseq (158) CGTTCCAACCACGTCTTCAAAGCAAGTGGATTGATGTGATATCTCCACTGACGTAAGGGATGACGCACAATCC-CACTATCCTTCGCAAGACCCTTCCTCTATATAAGGAAGTTCATTTCATT-TGGAGAGGACACG
Kefeng6_clipseq (289) CGTTCCAACCACGTCTTCAAAGCAAGTGGATTGATGTGATATCTCCACTGACGTAAGGGATGACGCACAATCC-CACTATCCTTCGCAAGACCCTTCCTCTATATAAGGAAGTTCATTTCATT-TGGAGAGGACACG
KMD_clipseq (289) CGTTCCAACCACGTCTTCAAAGCAAGTGGATTGATGTGATATCTCCACTGACGTAAGGGATGACGCACAATCC-CACTATCCTTCGCAAGACCCTTCCTCTATATAAGGAAGTTCATTTCATT-TGGAGAGGACACG
LL25_clipseq (1205) CGTTCCAACCACGTCTTCAAAGCAAGTGGATTGATGTGACATCTCCACTGACGTAAGGGATGACGCACAATCC-CACTATCCTTCGCAAGACCCTTCCTCTATATAAGGAAGTTCATTTCATT-TGGAGAGGACCCG
GTS40-3-2_clipseq (158) CGTTCCAACCACGTCTTCAAAGCAAGTGGATTGATGTGATACTTCCACTGACGTAAGGGATGACGCACAATCC-CACTATCCTTCGCAAGACCCTTCCTCTATATAAGGAAGTTCATTTCATT-TGGAGAGGACACG
MON1445_clipseq (158) CGTTCCAACCACGTCTTCAAAGCAAGTGGATTGATGTGATACTTCCACTGACGTAAGGGATGACGCACAATCC-CACTATCCTTCGCAAGACCCTTCCTCTATATAAGGAAGTTCATTTCATT-TGGAGAGGACACG
MON531-2_clipseq (158) CGTTCCAACCACGTCTTCAAAGCAAGTGGATTGATGTGATACTTCCACTGACGTAAGGGATGACGCACAATCC-CACTATCCTTCGCAAGACCCTTCCTCTATATAAGGAAGTTCATTTCATT-TGGAGAGGACACG
MON863_clipseq (158) CGTTCCAACCACGTCTTCAAAGCAAGTGGATTGATGTGATACTTCCACTGACGTAAGGGATGACGCACAATCC-CACTATCCTTCGCAAGACCCTTCCTCTATATAAGGAAGTTCATTTCATT-TGGAGAGGACACG
OXY235_clipseq (700) CGTTCCAACCACGTCTTCAAAGCAAGTGGATTGATGTGATATCTCCACTGACGTAAGGGATGACGCACAATCCGCACTATCCTTCGCAAGACCCTTCCTCTATATAAGGAAGTTCATTTCATTGTGGAGAGGACACG
M13F_clipseq (1) -------------------------------------------------GACGTAAGGGATGACGCACAA-------------------------------------------------------------------
M13P_clipseq (1) ------------------------------------------------------------------------CCCACTATCCTTCGCAAGACCCTTCC---------------------------------------
M13R_clipseq (1) ----------------------------------------------------------------------------------------------------------AGGAAGTTCATTTCATT-TGGAGAGG-----
M14F_clipseq (1) --------CCACGTCTTCAAAGCAAGTGG------------------------------------------------------------------------------------------------------------
M14P_clipseq (1) -----------------------------------------TCTCCACTGACGTAAGGGATGACGCA----------------------------------------------------------------------
M14R_clipseq (1) -----------------------------------------------------------------------------------------------------------GGAAGTTCATTTCATT-TGGAGAGGA----
Consensus (1212) CGTTCCAACCACGTCTTCAAAGCAAGTGGATTGATGTGATATCTCCACTGACGTAAGGGATGACGCACAATCC CACTATCCTTCGCAAGACCCTTCCTCTATATAAGGAAGTTCATTTCATT TGGAGAGGACACG

e

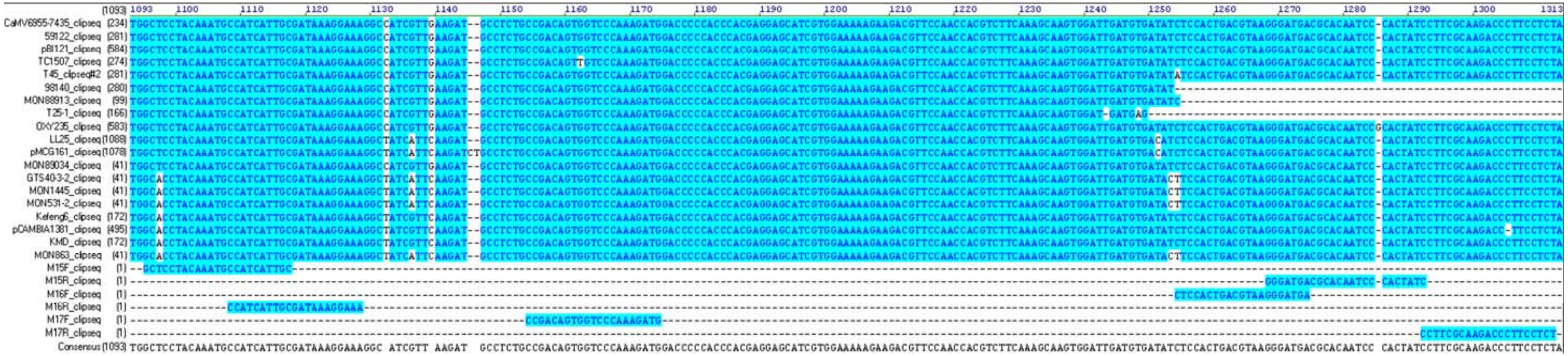
(1093) 1093 1100 1110 1120 1130 1140 1150 1160 1170 1180 1190 1200 1210 1220 1230 1240 1250 1260 1270 1280 1290 1300 1313
CaMV6955-7435_clipseq (234)
59122_clipseq (281)
pBI121_clipseq (584)
TC1507_clipseq (274)
T45_clipseq#2 (281)
98140_clipseq (280)
MON88913_clipseq (99)
T25-1_clipseq (166)
OXY235_clipseq (583)
LL25_clipseq (1088)
pMCG161_clipseq (1078)
MON89034_clipseq (41)
GTS40-3-2_clipseq (41)
MON1445_clipseq (41)
MON531-2_clipseq (41)
Kefeng6_clipseq (172)
pCAMBIA1381_clipseq (495)
KMD_clipseq (172)
MON863_clipseq (41)
M15F_clipseq (1)
M15R_clipseq (1)
M16F_clipseq (1)
M16R_clipseq (1)
M17F_clipseq (1)
M17R_clipseq (1)
Consensus (1093) TGGCTCCTACAAATGCCATCATTGCGATAAAGGAAAGGC ATCGTT AAGAT GCCTCTGCCGACAGTGGTCCCAAAGATGGACCCCCACCCACGAGGAGCATCGTGGAAAAAGAAGACGTTCCAACCACGTCTTCAAAGCAAGTGGATTGATGTGATATCTCCACTGACGTAAGGGATGACGCACAATCC CACTATCCTTCGCAAGACCCTTCCTCTA

f

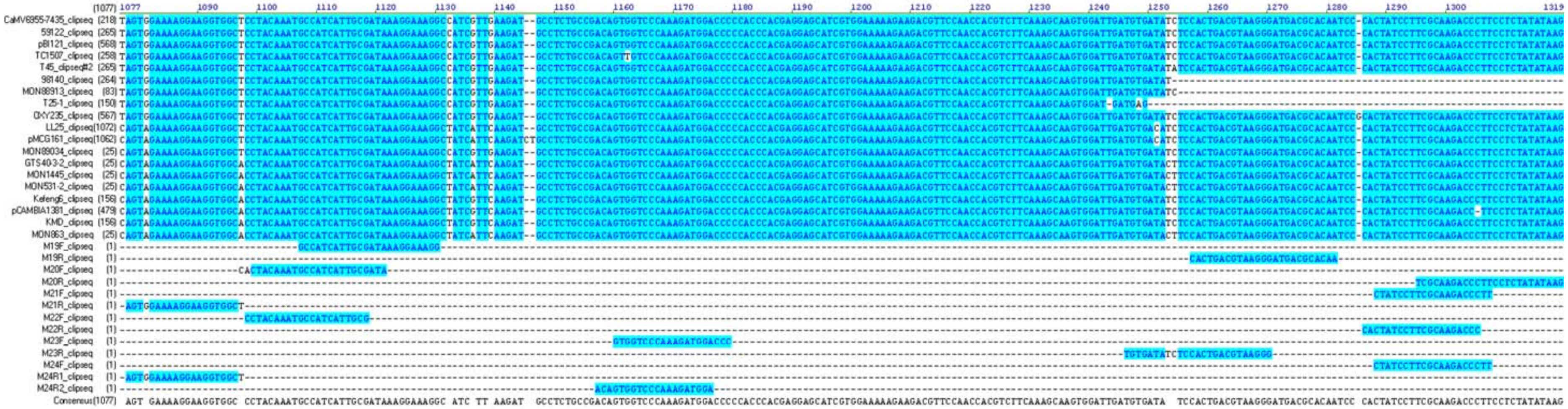

g

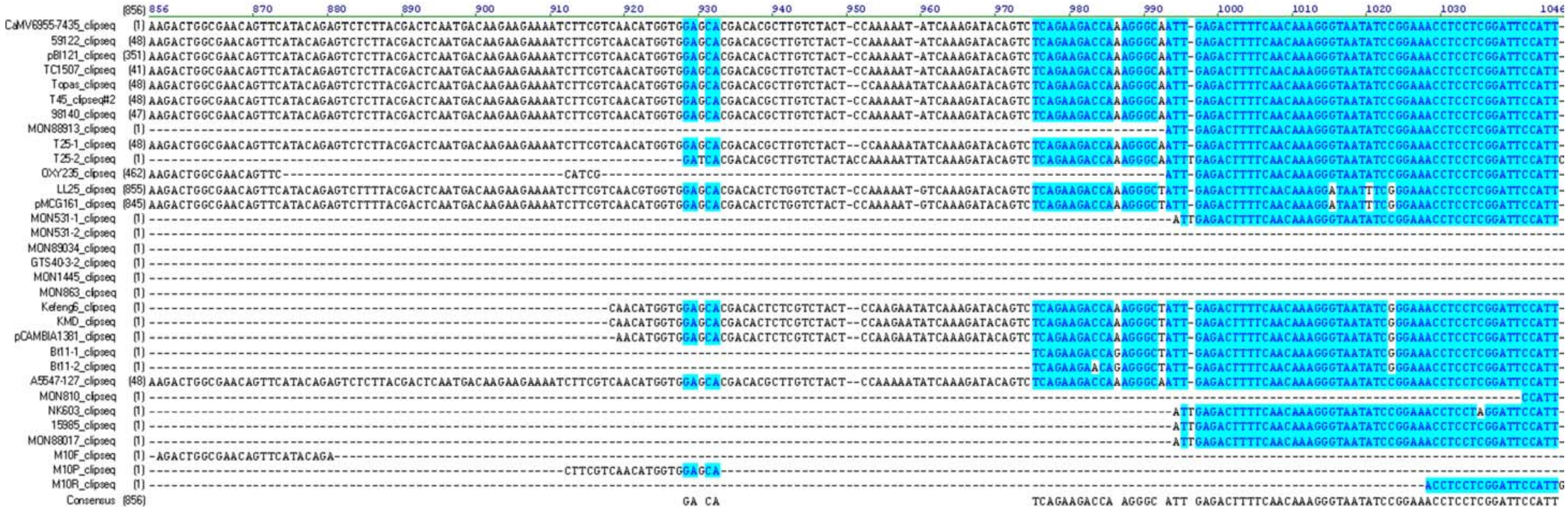
(856) 856 870 880 890 900 910 920 930 940 950 960 970 980 990 1000 1010 1020 1030 1046
CaMV6955-7435_clipseq (1)
59122_clipseq (48)
pBI121_clipseq (351)
TC1507_clipseq (41)
Topas_clipseq (48)
T45_clipseq#2 (48)
98140_clipseq (47)
MON88913_clipseq (1)
T25-1_clipseq (48)
T25-2_clipseq (1)
OXY235_clipseq (462)
LL25_clipseq (855)
pMCG161_clipseq (845)
MON531-1_clipseq (1)
MON531-2_clipseq (1)
MON89034_clipseq (1)
GTS40-3-2_clipseq (1)
MON1445_clipseq (1)
MON863_clipseq (1)
Kefeng6_clipseq (1)
KMD_clipseq (1)
pCAMBIA1381_clipseq (1)
Bt11-1_clipseq (1)
Bt11-2_clipseq (1)
A5547-127_clipseq (48)
MON810_clipseq (1)
NK603_clipseq (1)
15985_clipseq (1)
MON88017_clipseq (1)
M10F_clipseq (1)
M10P_clipseq (1)
M10R_clipseq (1)
Consensus (856) GA CA TCAGAAGACCA AGGGC ATT GAGACTTTTCAACAAAGGGTAATATCCGGAAACCTCCTCGGATTCCATT

h

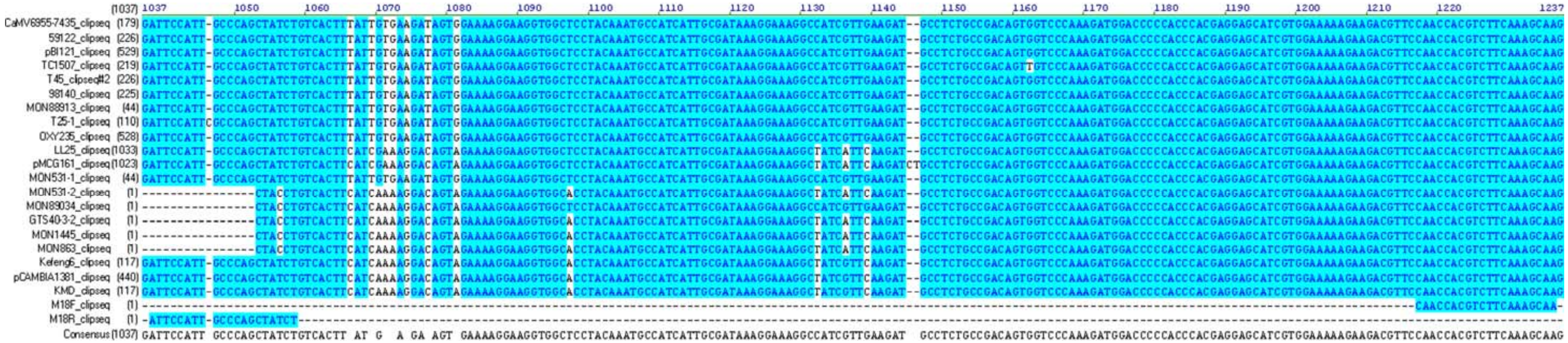

**Figure S1. Alignment of P35S sequences from the CaMV genome, multiple transgenic events, and binary vectors with SNPs, together with primer/probe sets. (a) alignment of M1 primer/probe set with P35S targets, (b) alignment of primer/probe sets of M3-M6 methods with P35S targets, (c) alignment of primer/probe sets of M8, M9 and M11 methods with P35S targets, (d) alignment of primer/probe sets of M13 and M14 methods with P35S targets, (e) alignment of primer pairs of M15-M17 methods with P35S targets, (f) alignment of primer pairs of M19-M24 methods with P35S targets, (g) alignment of M10 primer/probe set with P35S targets, (h) alignment of M18 primer pair with P35S targets.**
